# Assembling telomere-to-telomere genomes of *Fusarium oxysporum* f. sp. *lactucae* provides a roadmap for studying genome and phenotype evolution

**DOI:** 10.1186/s12864-026-12744-5

**Published:** 2026-04-07

**Authors:** Ningxiao Li, Jacob L. Steenwyk, Samuel O’Donnell, Emile Gluck-Thaler, Saben M. Kane, David M. Geiser, Frank N. Martin

**Affiliations:** 1 Crop Improvement and Protection Research Unit, United States Department of Agriculture – Agricultural Research Service, Salinas, CA 93905 USA; 2 Department of Plant Pathology and Environmental Microbiology, The Pennsylvania State University, University Park, PA 16802 USA; 3 Howard Hughes Medical Institute and the Department of Molecular and Cell Biology, University of California, CA 94720 Berkeley, USA; 4 Department of Plant Pathology, University of Wisconsin-Madison, Madison, WI 53706 USA; 5 Wisconsin Institute for Discovery, Madison, WI 53706 USA

**Keywords:** *Fusarium oxysporum* f. sp. *lactucae*, Fusarium wilt, Telomere-to-telomere, Accessory chromosomes, Transposable elements, Effectors, *Secreted in Xylem* (*SIX*), Comparative genomics, Genome evolution

## Abstract

**Background:**

Accessory genome regions of plant pathogenic fungi, which are highly variable and consist of niche-adaptive genes, play a crucial role in shaping host-specific interactions but are notoriously difficult to assemble. *Fusarium oxysporum* causes some of the world’s most economically devasting diseases, however, understanding how it interacts with its host is hindered by challenges in assembly of accessory genome regions/chromosomes, even with long read sequencing technologies. *F. oxysporum* f. sp. *lactucae* (FOLac) races 1 and 4 possess highly similar core genomes but cause distinct virulence phenotypes on specific lettuce cultivars. The availability of fully assembled genomes for the two races is needed to advance our understanding of the genetic basis of pathogenicity and the evolutionary processes underlying the diversification of FOLac and other *F. oxysporum* pathogens.

**Results:**

We developed an assembly workflow for generating gapless, telomere-to-telomere (T2T) complete genome assemblies for FOLac races 1 and 4. The T2T assemblies allowed for the identification of 16 chromosomes (5 accessory) and 20,616 predicted genes for race 1 and 19 chromosomes (8 accessory) and 20,292 predicted genes for race 4. Comparative genomics revealed major structural differences in their accessory genome regions, including genome rearrangement and large-scale chromosome duplication, with results suggesting transposable elements as the main drivers of those genomic changes. The analysis of *Secreted in Xylem* (*SIX*) effector gene profiles uncovered a similar presence/absence pattern among FOLac races 2–4, distinguishing them from race 1. Searches for genes unique to each race resulted in the identification of 687 race 1- and 536 race 4-specific genes. Assembly and genomic features comparing T2T to contig-level Illumina assemblies showed that 17–23% of genome sizes and ~ 10% of predicted genes were missing from Illumina assembly, mostly within accessory genome regions.

**Conclusions:**

T2T assemblies revealed large-scale differences in accessory genome structure and content between two otherwise highly similar pathogenic races. These differences provide a framework for understanding evolutionary processes that led to the diversification of pathogens within *F. oxysporum* on a fine evolutionary timescale, the identification of genes that may be responsible for host-pathogen interaction, and the identification of race-specific sequences useful for diagnostics.

**Supplementary Information:**

The online version contains supplementary material available at 10.1186/s12864-026-12744-5.

## Background

*Fusarium oxysporum* is a globally distributed species that includes important agents of wilt disease, but also strains that are endophytic/presumably nonpathogenic, and saprophytes. Collectively, plant pathogenic strains of *F. oxysporum* have a remarkably broad host range, causing vascular wilts as well as root and crown rots on over one hundred agronomically important plant species. While host diversity is broad at a species level, individual *F. oxysporum* pathogens usually display a high degree of host specificity, causing disease in one or a few related plant species [1, 2]. Pathogenic strains that infect the same plant host are grouped into the same *forma specialis* (f. sp.). More than 150 different *formae speciales* (ff. spp.) have been reported, with 106 of them having been well characterized [3, 4]. Some ff. spp. are further divided into races based on virulence to a set of differential host genotypes and, in some cases, based on known resistance genes in these hosts [3]. Races have been described in 25 ff. spp [3]., and new races arise frequently. A well-studied example is *F. oxysporum* f. sp. *lycopersici*, which had two successive resistance-breaking races emerge within 12 years of resistance deployment, as a result of coevolution between the pathogen and resistant tomato cultivars [5].

With very few exceptions where a single f. sp. is confined in a monophyletic lineage (such as f. sp. *ciceris* [6, 7]), *F. oxysporum* ff. spp. tend to be non-monophyletic [8, 9], consistent with independent evolutionary origins of pathogenicity on a given host and unique evolutionary patterns of different ff. spp. within *F. oxysporum*. With the origin of *F. oxysporum* being possibly as recent as ~ 0.5 mya [10], this diversification appears to have occurred very recently. Genetic determinants of host-specificity, including effector genes, are predominantly present in transposon-rich “accessory” chromosomes (ACs), also known as lineage-specific or pathogenicity chromosomes [11]. *F. oxysporum* has been reported to possess one or several ACs that are highly variable and harbor unique sequences that are absent in other *F. oxysporum* strains, except those that share the same host. This is in sharp contrast to core chromosomes (CCs), which are essential and conserved among all strains within *F. oxysporum*. The most studied effector genes in *F. oxysporum* are the *Secreted in Xylem (SIX)* genes, which were first identified in *F. oxysporum* f. sp. *lycopersici* and encode small, cysteine-rich proteins that are secreted into the xylem sap of tomato plants during infection [12–14]. Since then, 14 *SIX* genes (*SIX1-14*) and variability of them among different isolates and races within a single f. sp. have been reported in numerous *F. oxysporum* ff. spp [15–19]., with multiple studies illustrating the role of different *SIX* genes in conferring virulence and determining host specificity in several pathosystems [20, 21].

Fusarium wilt of lettuce, caused by *F. oxysporum* f. sp. *lactucae* (FOLac), has emerged as a major disease in lettuce growing areas, posing a significant threat to lettuce cultivation worldwide. Symptoms include stunting of growth, chlorosis of leaves progressing to leaf death and eventual death of the plant; when the stem is cut open vascular discoloration is often seen. The pathogen FOLac consists of four known races (races 1, 2, 3 and 4) that differ in virulence patterns on resistant or susceptible lettuce cultivars (for example, race 1 and 4 can be identified by differential virulence on cultivars, such as Lomeria and Romabella), with distinct geographic distributions. Race 1 is the most widespread and occurs in most global regions where lettuce is grown, including Japan [22, 23], the United States [24], Taiwan [25], Iran [26], Brazil [27], Argentina [28], and countries in Europe [29–32]. Races 2 and 3 have very restricted distribution and are found in Japan [23, 33] and Taiwan [25]. A new race (race 4), which is highly virulent on some race 1-resistant cultivars, was first detected in the Netherlands (Gilardi et al. [34]), where Fusarium wilt of lettuce was not previously reported. Since then, race 4 has spread rapidly to other parts of Europe, including Belgium [35], the UK [36], Italy [37], and Spain [38].

While FOLac races 2 and 3 are phylogenetically distinct from races 1 and 4 [25, 34, 39], all FOLac race 1 and race 4 isolates obtained from a worldwide collection were placed in a single clade based on sequences of 41 full-length, orthologous genes, with the two races being indistinguishable (D. Geiser, unpublished). This finding suggests recent clonal origin of FOLac race 1 and 4 pathogenicity. Using a *k*-mer-based approach that analyzes sequence variation directly from raw reads, FOLac races 1 and 4 can be further divided into two sub-clades, with varying levels of sequence divergence among individual isolates within each race [40]. It is speculated that intra-race genetic diversity within the race 1 and race 4 populations may contribute to the emergence of new variants or races of FOLac. This has been manifested in recent years, where novel pathogenic variants of FOLac race 1 were reported in California, with one variant (VSP-0916) exhibiting high aggressiveness on the race 1-resistant variety Costa Rica No.4, while another variant (Fol621) became less virulent on susceptible cultivars [41]. Similarly, an emerging FOLac race 4 + was detected in several farms in Belgium where FOLac race 4 intermediate resistant cultivars showed wilting and growth reduction one year after commercialization [42].

To better understand the evolutionary processes responsible for the emergence and diversification of the FOLac pathogen, a reference genome of FOLac that is fully assembled is critical to the research community. Although the disease has been reported for nearly half a century, the first relatively complete genome of FOLac was not released until recently [43]. Based on comparative genomic analyses between FOLac races 1 and 4, Bates et al. [43] showed that FOLac race 4 has a larger genome than FOLac race 1, with a lack of synteny between their accessory genome regions. Additionally, Bates et al. [43] posited that FOLac race 4 did not evolve directly from FOLac race 1, but both races inherited their accessory genomes from a common ancestor, which then underwent rearrangement/recombination events, accompanied by the acquisition of accessory regions via horizontal chromosome transfer. However, because these published FOLac assemblies are at the contig-level, with fragmented CCs and ACs, it is necessary to reconstruct a complete genome for FOLac races 1 and 4 to provide a more complete insight into the mechanisms underlying genome evolution that led to the diversification and host specialization of the pathogen.

Here, we present gapless, telomere-to-telomere (T2T) complete genome assemblies for FOLac races 1 and 4. In doing so, we developed a workflow for generating T2T assemblies and highlight the importance of visual inspection of read mapping to ensure the accuracy and correctness of the assembly. Comparative genomics between the two T2T assemblies revealed major structural differences in their accessory genome regions that may underlie genetic diversification between FOLac races 1 and 4. The T2T assemblies also enabled the characterization of large-scale chromosome duplication and the recognition of a specific type of transposable elements (TEs) that may be involved. A comprehensive transcriptome dataset of FOLac, which encompassed nine RNA-seq libraries generated under different growth conditions, was used to produce a complete genome annotation for the two T2T assemblies, leading to the identification of candidate genes that may be race-specific. Moreover, this study compared assembly and genomic features between the T2T assembly and two contig-level assemblies for the same isolates, offering researchers a new perspective in assessing their sequencing and assembly strategy.

## Results

### FOLac race 4 has an expanded genome with three more chromosomes than race 1

**Fig. 1:**
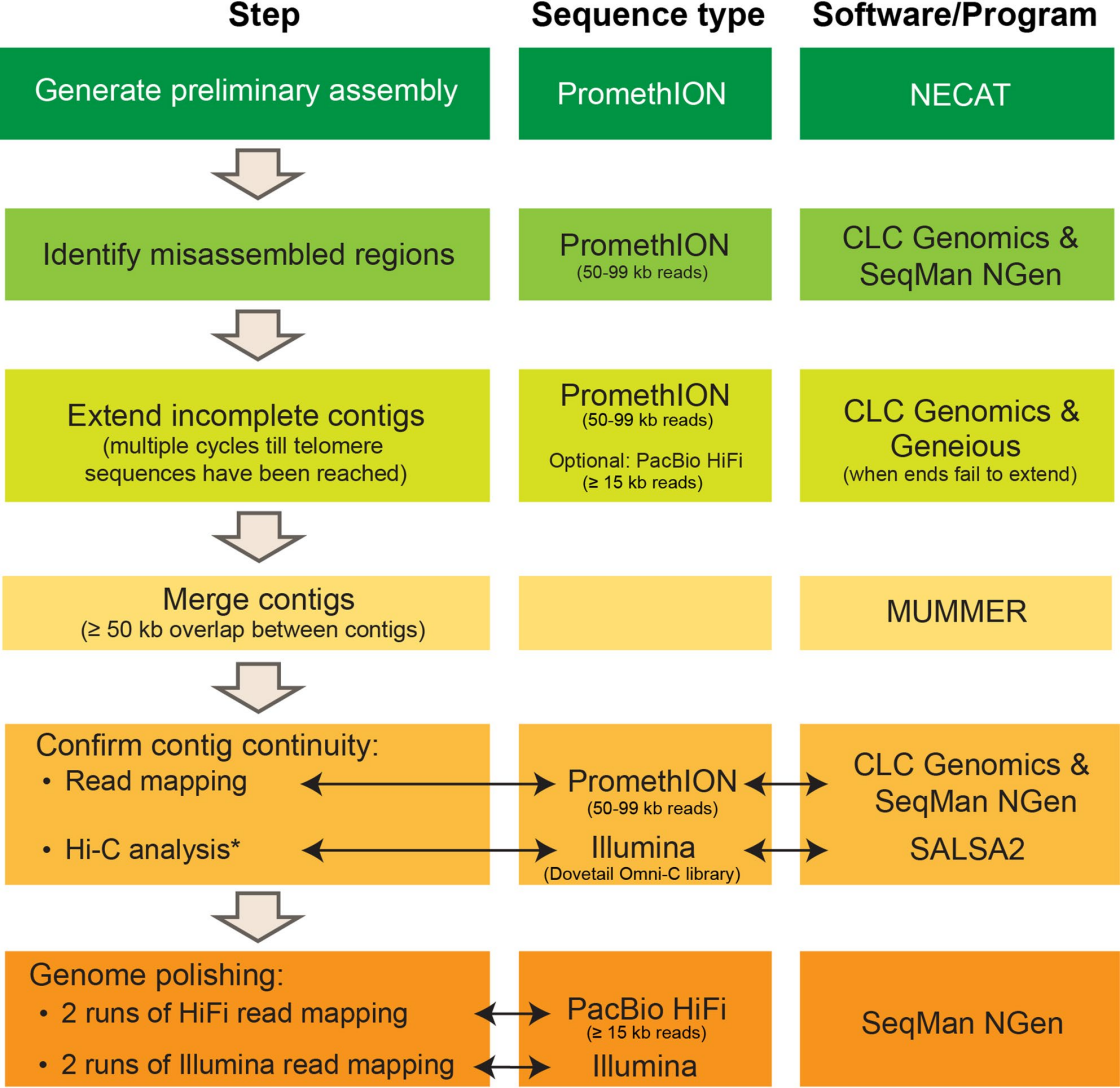
Flowchart depicting the assembly workflow to generate gapless, T2T complete genome assemblies for FOLac isolates JCP043 and AT141. Details of the workflow are delineated in Methods and Supplementary File S1. * Hi-C analysis was performed on JCP043 only

Two previous reported *F. oxysporum* isolates, JCP043 (FOLac race 1, from California [24, 44] and AT141 (FOLac race 4, from Spain [40]), were selected for long-read sequencing. The assembly workflow (Fig. 1) started with building a preliminary assembly using reads generated by Oxford Nanopore Technologies (ONT). Using default settings, the NECAT program assembled the ONT reads into 27 contigs for JCP043 and 25 contigs for AT141, some of which already had telomeric repeats (CCCTAA/GGGTTA) on one or both ends. The mitochondrial (mt) genome was identified from the assembly and deposited in GenBank (PX514187 and PX514188). By mapping 50–99 kb ONT reads to the NECAT contigs, mis-assemblies were visually identified based on a sharp decrease in read depth at the misassembly junction and no reads spanning the incorrectly assembled region (example shown in Supplementary Figure S1); reads less than 50 kb were not used to ensure reads would overlap repetitive sequences and their flanking regions, thereby reducing problems with mis-assembly. After the assembly inspection, the NECAT contigs were broken into 44 intermediate contigs for JCP043 and 46 contigs for AT141.

Iterative cycles of read mapping with 50–99 kb ONT reads followed by end extension were carried out on the intermediate contigs. In most cases, ONT reads alone were sufficient to extend the contigs to the telomeric region without taking extra steps (see detailed instructions in Methods and Supplementary File S1). The extended contigs with an overlap of greater than 50 kb and 95% sequence identity were joined to reconstruct full-length chromosomes. The telomeric end of the rDNA repeat region was completed through aligning 50–99 kb reads that contained rDNA sequences and flanking sequences. The rDNA copy number was estimated based on the depth of ONT read coverage relative to adjacent regions of the chromosome, which resulted in an estimated 90 copies for JCP043 and 144 copies for AT141. The final T2T assembly was evaluated for accuracy again by mapping 50–99 kb ONT reads to it, with visual inspection showing continuous and uniform read coverage throughout the chromosomes.

Due to the error rates (between 4 and 5% at the time of data generation) of ONT data, the T2T assembly was polished using a hybrid strategy involving two runs of PacBio HiFi read mapping followed by two runs of Illumina read mapping at high stringency (to reduce problems of degenerate bases flanking repetitive regions). Although Illumina reads exhibited great read accuracy, many regions in the genome, especially those in the ACs, did not have sufficient read depth required for performing error correction (Supplementary Table S1 and Supplementary Figure S2). Insufficient coverage was more pronounced in AT141 than JCP043, as the gapped genomic regions totaled 2.17 Mb in AT141 compared to 156 kb in JCP043 (Supplementary Table S1). As much as 10.5% of chromosome 16 in AT141, mainly in the 5’end of the chromosome (nonrepetitive, 280 kb), was depleted of Illumina reads. In contrast, HiFi reads had a uniformly high read coverage (> 100× coverage with reads ≥ 15 kb) across the entire genome (Supplementary Figure S2). However, single-base sequence errors, including SNPs and indels, could still be detected in the HiFi-corrected assembly; therefore, additional error correction using Illumina reads was carried out under high stringency. The accuracy of the polished assembly of JCP043 was further confirmed with high-throughput chromosome conformation capture (Hi-C) analysis, and the result showed no mis-assembly (Supplementary Figure S3). The number of chromosomes was also validated by centromeric interaction regions detected in the Hi-C contact map, with each contig represented as a single chromosome.

The final assemblies were gapless and T2T complete, with JCP043 (race 1) and AT141 (race 4) assembled into 16 and 19 chromosomes, respectively, and the AT141 genome size being 6% or 3.86 Mb greater than that of JCP043 (Table 1). The benchmarking universal single-copy ortholog (BUSCO) analysis was performed to assess the genome completeness. Of the 4,494 BUSCOs searched (library hypocreales_odb10), 98.9% and 98.5% were detected as complete and single copy in JCP043 and AT141, respectively (Table 1), which had similar levels of completeness compared to other published FOLac genomes (98.8% for AJ520 and 98.7% for AJ516 [43]).

**Table 1:** Comparison of assembly and genomic features between T2T, Illumina, and NECAT assemblies of FOLac race 1 isolate JCP043 and race 4 isolate AT141

	JCP043			AT141		
	T2T1	Illumina	NECAT	T2T1	Illumina	NECAT
Assembly features						
Genome size (Mb)	**64.64**	58.59	63.73	**68.50**	53.58	67.10
Read depth	**204×** ^**2**^	146×	204 × ^**2**^	**176×** ^**2**^	182×	176 × ^**2**^
GC (%)	**47.61**	49.34	47.42	**47.67**	48.17	47.62
N50 (Mb)	**4.63**	0.14	4.31	**4.25**	0.10	4.18
L50	**6**	112	6	**7**	148	7
# of contigs	**16**	5,405	27	**19**	5,771	25
# of T2T contigs	**16**	0	5	**19**	0	12
Longest contig (Mb)	**6.77**	0.91	7.07	**6.82**	0.54	6.82
BUSCO (complete, single-copy)	**98.9%**	98.8%	98.9%	**98.8%**	98.6%	98.8%
Core genome size (Mb)	**48.49**	46.47^3^	48.09^3^	**48.98**	43.98^3^	47.44^3^
Accessory genome size (Mb)	**16.15**	7.62^3^	15.51^3^	**19.52**	7.38^3^	15.61^3^
Genomic features						
Total genes	**20**,**616**	18,811	20,517	**20**,**292**	18,320	20,185
Total transcripts	**22**,**319**	20,120	21,983	**21**,**918**	19,601	21,572
Total proteins	**22**,**083**	-	-	**21**,**680**	-	-
Mean gene length	**1**,**746 bp**	-	-	**1**,**738 bp**	-	-
Mean exon length	**630 bp**	-	-	**623 bp**	-	-
Repeat regions	**17.64%**	6.46%	18.29%	**20.39%**	4.86%	19.97%
GO terms	**12**,**293**	-	-	**12**,**163**	-	-
PFAM	**13**,**204**	-	-	**13**,**081**	-	-
CAZYmes	**700**	671	700	**705**	659	702
* Secreted CAZYmes*	**331**	312	331	**337**	301	325
SM clusters	**65**	65	65	**67**	64	67
* SM genes*	**1**,**051**	1,036	1,051	**1**,**028**	1,004	1,026
Secreted proteins	**1**,**433**	1,316	1,422	**1**,**451**	1,303	1,414
* Effector proteins*	**605**	559	600	**574**	526	573
* Mimp effectors*	**94**	90	94	**82**	78	82
Race-specific genes	**687**	518	681	**536**	437	513
* Secreted CAZYmes*	**4**	3	4	**2**	2	2
* Effector proteins*	**14**	9	14	**8**	7	8
* Mimp effectors*	**5**	4	5	**3**	3	3
* SM genes*	**3**	2	3	**6**	5	6

^1^Numbers associated with T2T assemblies are highlighted in bold

^2^Read coverage of T2T and NECAT assemblies was calculated by mapping 50–99 kb ONT reads to the assembly for improved mapping accuracy

^3^Core and accessory genome sizes of Illumina and NECAT assemblies were calculated based on the total length of aligned contigs to the core and accessory genome regions of their corresponding T2T assembly

- indicates comparison was not performed on the specific feature.

### Massive genome rearrangement in FOLac accessory chromosomes

**Fig. 2:**
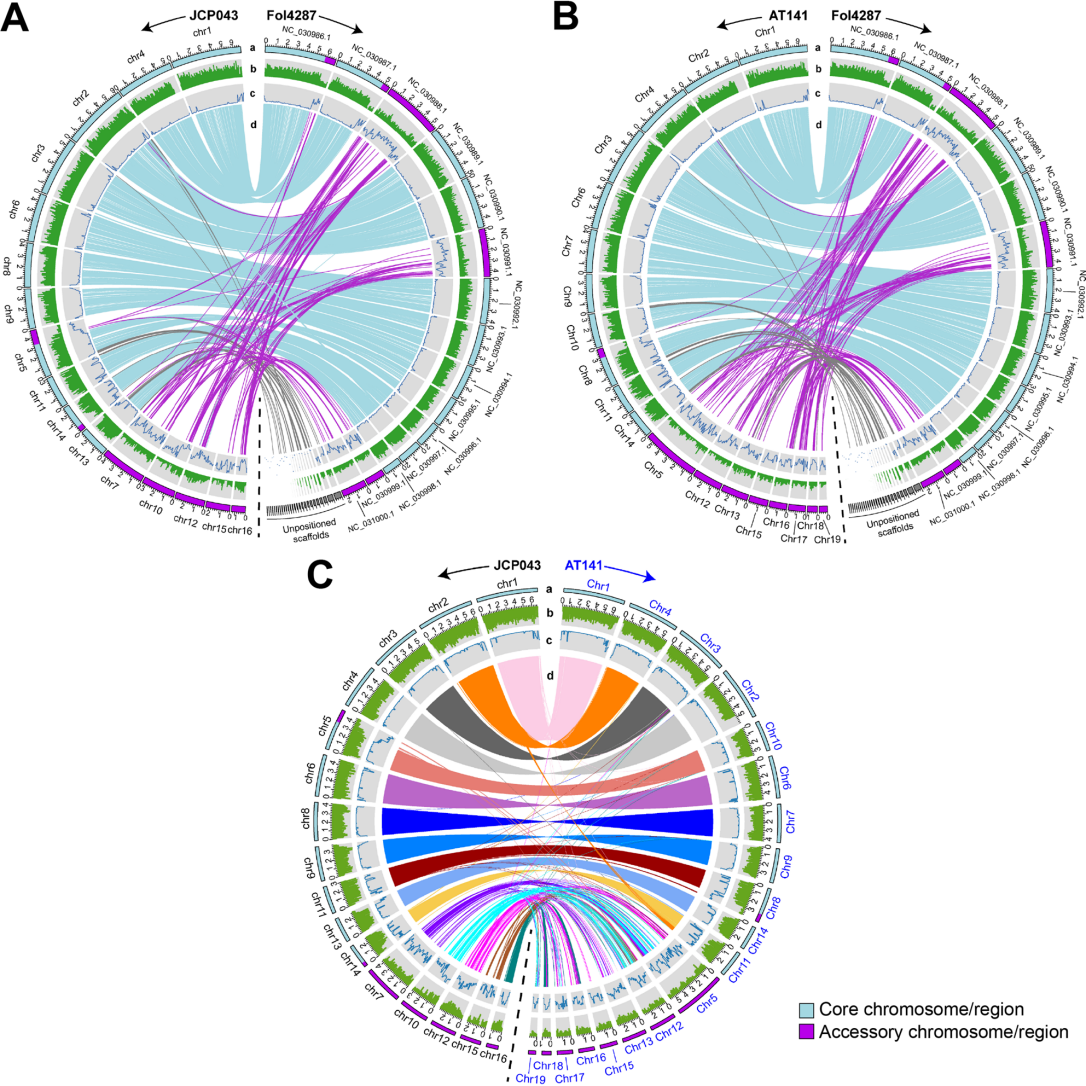
Synteny of chromosomes between FOLac race 1 JCP043, race 4 AT14 and *F. oxysporum* f. sp. *lycopersici* Fol4287. **A** Circos plot of JCP043 vs. Fol4287. **B** Circos plot of AT141 vs. Fol4287. **C** Circos plot of JCP043 vs. AT141. Order of tracks from outward to inward (a-d): a: Cyan and purple boxes represent core and accessory chromosomes/genome regions, respectively; b: The abundance of annotated genes in each 100 kb window. Black tic markers occur every 200 kb on the outside of the ideogram; c: The proportion of each 100 kb window covered by transposable elements; d: Lines that connect the chromosomes between the two genomes represent similar regions greater than 10 kb in size

Comparison of each of the two T2T assemblies to that of Fol4287 (f. sp. *lycopersici* race 2), the reference genome of *F. oxysporum* due to its nearly complete genome featuring 11 CCs and 5 ACs, revealed that (i) each of the two FOLac isolates had 11 CCs that were highly syntenic with the 11 CCs of Fol4287 (Fig. 2A and B), and exhibited common features associated with CCs, including high gene density and low abundance of TEs; (ii) JCP043 had five ACs while AT141 featured eight ACs that did not show clear synteny with the Fol4287 genome, and exhibited accessory-like appearance (gene-sparse and TE-rich); (iii) the accessory genome regions of JCP043 also included a 1.52-Mb terminal segment of chromosome 5 and a 0.53-Mb segment of chromosome 14, whereas a 0.96-Mb terminal segment of chromosome 8 in AT141 was part of its accessory genome. It was worth noting that many of the unplaced scaffolds of Fol4287 were aligned with several CCs of JCP043 and AT141, primarily to the sub-telomeric regions.

The collinearity analysis of JCP043 and AT141 showed that the 11 CCs were highly syntenic between the two isolates, with 99%-100% sequence identity except the accessory regions located on chromosomes 5 and 14 of JCP043 and chromosome 8 of AT141, which appeared to be unique sequences to each race (Fig. 2C). This analysis also identified one chromosomal translocation event, in which chromosome 11 of AT141 appeared to be translocated to two different chromosomes in JCP043, with nearly 80% of the chromosome landing on chromosome 14 of JCP043 and the remaining 20% fused to the terminus of chromosome 2 (Fig. 2C). This translocation event was also observed in the synteny plot between JCP043 and Fol4287 (Fig. 2A).

**Fig. 3:**
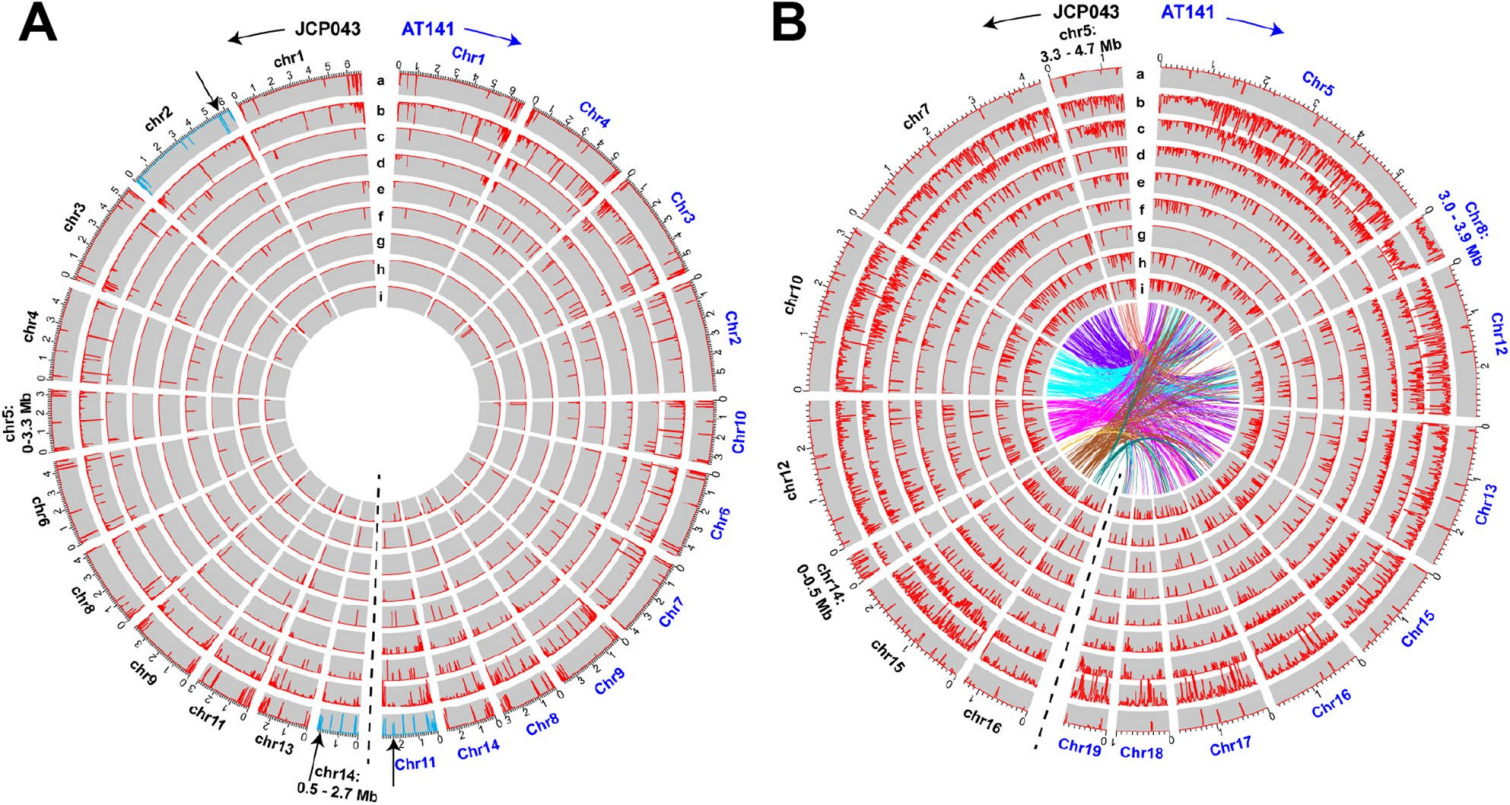
Distribution of different classes of transposable elements in the core and accessory chromosomes/regions of JCP043 and AT141. **A** Core chromosomes/regions of JCP043 and AT141. The breakpoint on chromosome 11 of AT141 and the two fusion points on chromosomes 2 and 14 of JCP043, which are highlighted in black arrows, overlap with *Gypsy/DIRS1* elements (highlight in blue). **B** Accessory chromosomes of JCP043 and AT141. Lines in the center represent shared regions (≥ 2 kb in size and ≥ 85% of sequence identity) within the large unique regions that are unshared between the two FOLac isolates. Repeat-masked accessory chromosomes are used in this analysis. Order of tracks from outward to inward (a-i): a: *Gypsy/DIRS1*, b: uncharacterized repetitive sequence, c: *hAT-Restless*, d: *Tc1/Mar*-*Fot1*, e: *MULE-MuDR*, f: *PiggyBa*c, g: *Helitron*, h: *Ty1/Copia*, and i: *Tad1*

Comparison of the ACs between JCP043 and AT141 showed an absence of large-scale synteny between the two isolates, but revealed widespread presence of smaller similar sequences, ranging from 10 to 75 kb in size and 92.99–98.92% sequence identity, between them in a non-colinear order (Fig. 2C), which suggests massive genome rearrangements may have occurred in their ACs. One exception to this was chromosome 16 of JCP043, which appeared to have two large fragments of DNA, 480 and 580 kb in size, syntenic with chromosomes 16 and 18 of AT141, respectively. To get a better understanding of how dissimilar the unique sequences (defined by the lack of homology in any 10-kb windows) were between the two isolates, the two sets of ACs were compared at 2-kb resolution (slightly above the average gene length). The analysis, which was performed on repeat-masked ACs to avoid the detection of overwhelmingly abundant DNA repeats, showed that 32% of those unique segments were shared between the two isolates (Fig. 3).

To determine whether the ACs of JCP043 are conserved in race 1 while variable to race 4, we conducted synteny analysis with six published contig-level FOLac genomes (three race 1 and three race 4 isolates [43]). The result showed that race 1 isolate JCP043 displayed high levels of AC synteny with previously published race 1 isolates (AJ520, AJ718 and AJ865), whereas most of the ACs in JCP043 were not syntenic with the race 4 isolates (AJ516, AJ592 and AJ705) (Supplementary Figure S4A). Likewise, the ACs of race 4 isolate AT141 were syntenic with the three race 4 isolates but not with race 1 (Supplementary Figure S4B).

### DNA repeats likely contribute to the expanded genome of AT141

**Fig. 4:**
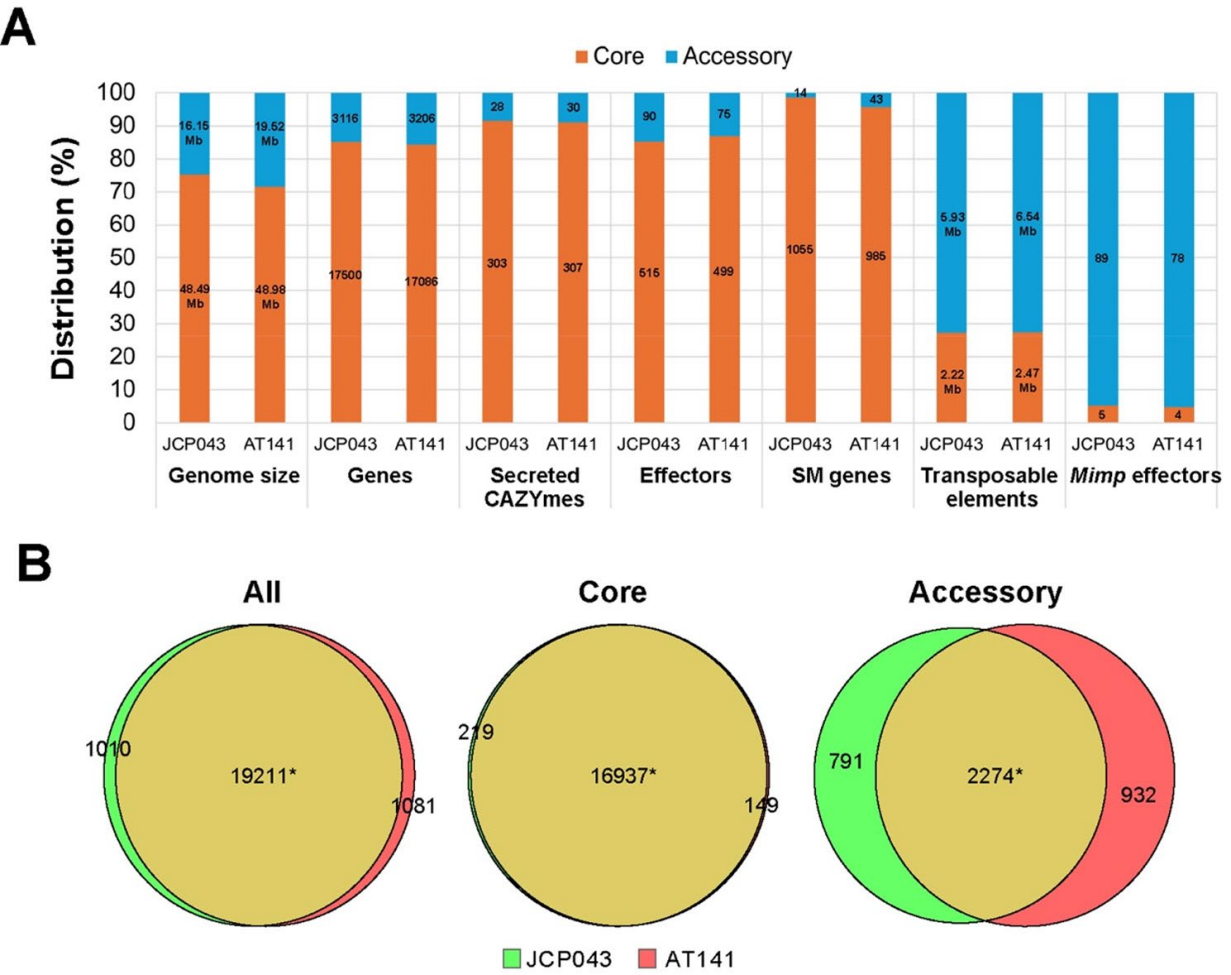
Genomic features of JCP043 and AT141. **A** Distribution of genomic features in the core (orange) vs. accessory (blue) genome regions of JCP043 and AT141, including genes that encode secreted CAZYmes (carbohydrate-activate enzymes), effectors, SM (secondary metabolite) genes, transposable elements, and *mimp-*associated effectors. Numbers in the bars indicate total length or number of genes. **B** Venn diagrams depicting shared and unique genes between JCP043 and AT141 genomes. The numbers marked with asterisks are based on the gene set of AT141. As for JCP043, the total shared genes for all, core and accessory genome regions are 19,606, 17,281, and 2325 respectively, due to the fact that some of the shared genes are in multiple copies in JCP043

About 18% and 20% of the JCP043 and AT141 genomes, respectively (Table 1), was identified as repetitive sequence, including DNA transposons (*hAT*, *Tc1-IS630-Pogo*, *MULE-MuDR*, *PiggyBa*c and *Helitron*), long terminal repeat (LTR) retrotransposons (*Gypsy/DIRS1* and *Ty1/Copia*), long interspersed nuclear elements (LINEs) retrotransposons (*Tad1* and *RTE*), as well as simple repeats and satellites (Supplementary Table S2). Among the different classes of TEs, *hAT* represented the largest in length and ranked the highest in copy number, followed by *Gypsy/DIRS1* and *Tc1-IS630-Pogo.* Based on the density plots (Fig. 3), ACs were enriched in different types of TEs, accounting for more than 75% of the characterized TEs in the entire genome (Fig. 4). While most of the repeat classes were similar in size between the two genomes, the AT141 genome contained a significantly greater amount of uncharacterized repetitive sequence, totaling 4.34 Mb in size compared to 2.61 Mb in JCP043, accounting for 44.8% of the overall genome size difference between the two isolates. The uncharacterized repeats were widely distributed among the ACs, especially in the accessory segment of chromosome 8 in AT141 (Fig. 3). Sequences of the uncharacterized repeats are provided in Supplementary File S2.

While most of the TEs were largely less abundant in CCs, we noticed that 75% of *Gypsy/DIRS* elements were associated with CCs (Fig. 3). To determine whether there is any association between *Gypsy/DIRS1* and the aforementioned chromosomal translocation in JCP043, we marked the breakpoint (located on chromosome 11 of AT141) and two fusion points (located on chromosomes 2 and 14 of JCP043) on the TE density plot. It was found that the breakpoint and one of the fusion points (on chromosome 2 of JCP043) overlapped with a *Gypsy/DIRS1* element while the other fusion point (on chromosome 14 of JCP043) resided 15 kb upstream of three *Gypsy/DIRS1* elements (Fig. 3A).

### Large-scale chromosome duplication is prevalent in FOLac accessory chromosomes

**Fig. 5:**
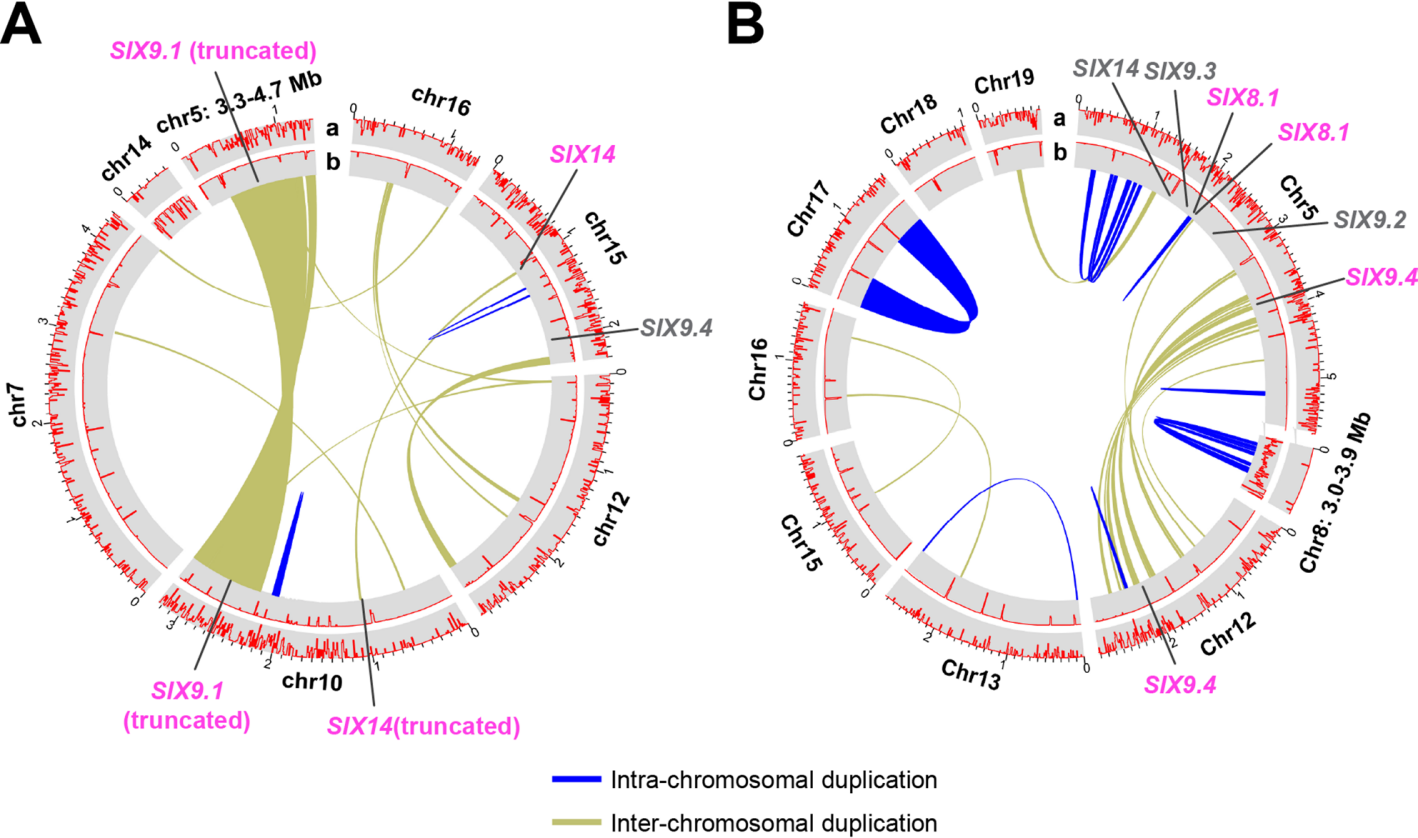
Large-scale chromosome duplication in JCP043 and AT141 and the distribution of *SIX* genes among accessory chromosomes/genome regions. **A** Accessory chromosomes/genome regions of JCP043. **B** Accessory chromosomes/genome regions of AT141. The accessory regions of each genome were aligned against themselves using the nucmer package (nucmer –maxmatch; -L 20000) to identify highly similar regions. Lines in the center represent homologous regions (≥ 2 kb in size and ≥ 85% of sequence identity). The distribution of *hAT-Restless* and *Gypsy/DIRS1* is shown on track a and b, respectively. *SIX* genes located within the duplicated regions are marked in pink while single-copy *SIX* genes are noted in grey

Besides DNA repeats, we explored the possibility of segmental AC chromosome duplication contributing to the larger genome size of AT141, which showed an AC count of eight in comparison to the five observed in JCP043 assembly. To study this, pair-wise comparison of individual ACs of AT141 were conducted to identify similar regions greater than 20 kb (to avoid the detection of overwhelmingly abundant DNA transposons, some of which could extend up to 12 kb). The analysis resulted in the recognition of numerous intra- and inter-chromosomal duplications in all the ACs of AT141 (Fig. 5) that ranged in size from 20 kb to 500 kb, except for chromosome 18, which did not show any segmental duplication. Notably, we identified two nearly identical, 500-kb inverted repeats located on both ends of chromosome 17 (Fig. 5), flanking a 700-kb non-repetitive region. This duplication, along with a few others, might be facilitated by *Gypsy/DIRS1* retrotransposons that flank the duplicated regions (Fig. 5). In addition, a heavy presence of *hAT-Restless* transposons was detected surrounding some of the other duplicated regions.

While the frequency of intra-chromosomal duplication was markedly less in JCP043 compared to AT141, inter-chromosomal duplications were frequently found in the ACs of JCP043, including a 1-Mb region that was nearly identical between chromosomes 5 and 10 (Fig. 5A). Similar to AT141, we found the association of *Gypsy/DIRS1* with the boundaries of this large, duplicated region.

Overall, given that the total size of duplicated regions was nearly identical between the two genomes (1.43 Mb for JCP043 vs. 1.52 Mb for AT141), it was reasonable to conclude that segmental chromosome duplication was not the major contributing factor for the expanded genome of AT141, but it may play an important role in the formation of new accessory chromosomes (i.e. chromosome 17) and altering structural organization of the genome.

### General putative effector proteins are distributed more in the core genome regions while *mimp*-associated effectors are enriched in the accessory genome regions

The genomes of JCP043 and AT141 harbored 20616 and 20292 predicted genes, respectively (Table 1), however, only 60% of them had GO annotations. Approximately 85% of the predicted genes were associated with the core genome regions while the remaining 15% were present in the accessory genome regions (Figs. 4A). Using the non-redundant gene sets for JCP043 and AT141, we identified 15980 pairs of putative orthologous genes. Not surprisingly, over 94% of them were associated with CCs and the co-linear order of genes between the two isolates has maintained within these chromosomes (Supplementary Figure S5A). The remaining 6% were widely dispersed among different ACs (Supplementary Figure S5B).

The JCP043 and AT141 genomes harbored similar numbers of several specific gene classes, respectively, including 331/337 extracellularly secreted carbohydrate-active enzymes (CAZYmes), 605/574 effector proteins, and 65/67 secondary metabolite (SM) gene clusters comprising 1051/1028 genes (Table 1). These genes were predominantly present in CCs, representing 85–98% of the gene reservoir (Fig. 4A and Supplementary Figure S5). Among the putative effector proteins identified, apoplastic effectors constituted 51–54% of the effector complement, followed by cytoplastic effectors (27–30%). The localization of the remaining effector proteins remained undetermined.

According to previous studies, many effector genes (e.g. *SIX* genes) in *F. oxysporum* are located within subregions enriched for TEs, and a miniature Impala (*mimp*) element, in particular, is always present in their promoters [45, 46]. The analysis of *mimp*-associated effectors identified 94 and 82 candidates in the genomes of JCP043 and AT141, respectively. Among them, 18 (for JCP043) and 11 (for AT141) *mimp* effectors overlapped with the effector proteins identified using the EffectorP pipeline. Nearly 95% of the candidate *mimp* effectors were associated with ACs (Fig. 4A), with chromosome 10 of JCP043 (*N* = 29) and chromosome 5 of AT141 (*N* = 35) harboring the most *mimp* effectors (Supplementary Figure S5B).

### Genes involved in intracellular pH homeostasis, regulation of transcription and mitochondria-nucleus signaling pathway are enriched in FOLac accessory chromosomes

We conducted gene ontology (GO) enrichment analysis to identify putative biological processes and molecular functions that are enriched in the ACs of both FOLac isolates. It turned out that their ACs were significantly enriched (corrected *p*-value < = 0.01) for biological processes involved in intracellular monoatomic ion and cation homeostasis, regulations of intracellular pH and DNA-binding transcription factor activity, and mitochondria-nucleus signaling pathway (Supplementary Table S3). Molecular functions, including various types of oxidoreductase activities and iron ion and heme-binding activities, were also enriched. In addition, the ACs of JCP043 were uniquely enriched for genes involved in lipid, peptide, and monocarboxylic acid catabolic processes, while genes involved in double-strand break repair DNA repair, regulation of nitrogen utilization, and long-chain fatty acid metabolic process were uniquely enriched in the ACs of AT141. Regarding the genes located within the duplicated regions in JCP043, they were enriched for two biological processes, including intracellular monoatomic ion and cation homeostasis and chromate transport. No GO terms were significantly enriched in the duplicated regions in AT141.

### *SIX9.4* and *SIX14* are conserved in FOLac while *SIX8* and *SIX9.1-9.3* are absent in race 1

Due to the lack of ability to identify all 14 *SIX* genes using the EffectorP and FoEC2 pipelines [47], we manually annotated the *SIX* gene complement in both T2T assemblies via BLAST. Moreover, we expanded the search to 53 publicly available FOLac genomes [40, 41, 43], including 33 race 1 isolates, 1 race 2 isolate, 1 race 3 isolate, 16 race 4 isolates, and 2 race 1 variants (Supplementary Table S4), to evaluate the correlation between the *SIX* gene complement and race structure. Additional long-read genome assemblies representing different phylogenetic lineages and clades outside of FOLac (Supplementary Table S4) were also included in this analysis to determine how similar their *SIX* genes are to those present in FOLac. Generally, FOLac carried three *SIX* genes, *SIX8*, *SIX9* and *SIX14*, with copy number and sequence variations at the intra and inter-race levels, which are described below. None of the remaining *SIX* genes were identified in FOLac.

#### SIX8

**Fig. 6:**
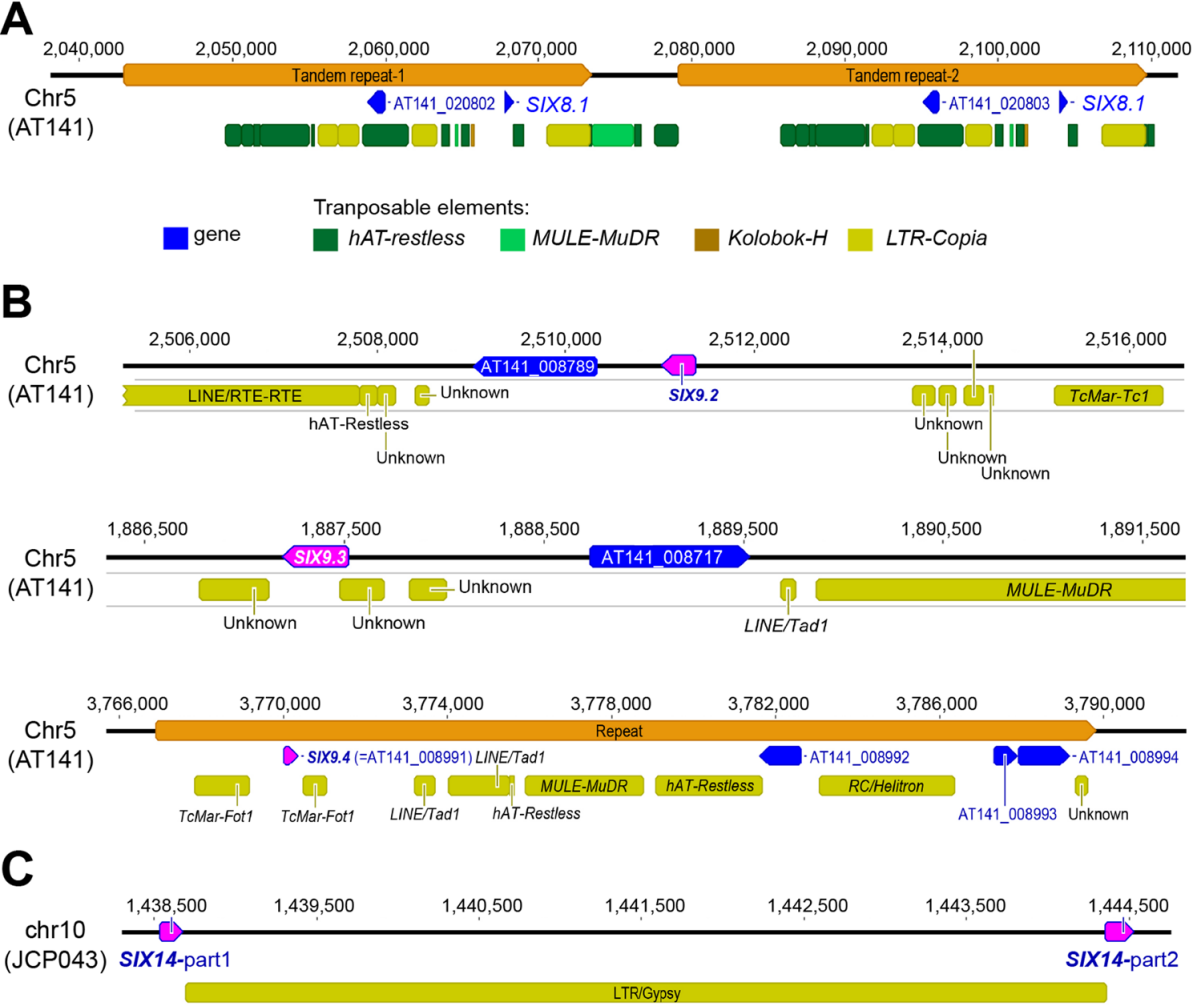
Schematic representations of the genomic regions harboring the two identical copies of *SIX8* (**A**), the three variants of *SIX9* (**B**), and the truncated version of *SIX14* (**C**) in the context of transposable elements

*SIX8* was absent in JCP043 and 35 other FOLac race 1 isolates (including the two race 1 variants), while two identical copies of *SIX8* were identified in AT141, located within a 30-kb, TE-rich, tandem repeat on chromosome 5 (Figs. 5B and 6A). Except for AT141 having two copies of *SIX8*, a single copy of *SIX8* was consistently identified in all the other FOLac race 4 isolates as well as FOLac races 2 and 3, with variation in the *SIX8* sequences among isolates resulting in two *SIX8* variants (*SIX8.1* and *SIX8.2*; Supplementary Figures S6 and S7). *SIX8* was also present in eight other unrelated *F. oxysporum* isolates, including *ff. spp. conglutinans*, *lycopersici*, *niveum*, and *sesame* (Supplementary Table S4). The *SIX8* gene phylogeny revealed that FOLac *SIX8.1* and *SIX8.2* were most closely related to f. sp. *conglutinans* (Supplementary Figure S7).

#### SIX9

A previous study showed that FOLac had four variants of *SIX9* (*SIX9.1-SIX9.4*), with differences in copy number and sequence variation between races 1 and 4 [43]. In our study, none of the FOLac race 1 isolates had *SIX9.1*, *SIX9.2*, and *SIX9.3*, except race 1 isolate AM163, which appeared to have one copy of *SIX9.2* in its Illumina assembly. However, the read mapping analysis indicated otherwise due to the extremely low read depth (less than 8×) on *SIX9.2* compared to the other *SIX* genes (Supplementary Figure S8). Two identical copies of truncated *SIX9.1* (54% in coverage), which had an uncharacterized type of DNA repeat present upstream, were identified in JCP043 (Fig. 5A). The truncated *SIX9.1* was conserved among all the FOLac race 1 isolates. FOLac races 2, 3 and 4 possessed all four variants of *SIX9*, but variation in copy numbers was observed among the FOLac race 4 isolates (Supplementary Table S4). *SIX9.4* appeared to be the only copy that was consistently identified from all four races, with no sequence variation among isolates (Supplementary Figure S7). Based on their location on the T2T assembly of AT141, the three variants of *SIX9* (*SIX9.2*, *SIX9.3*,* and SIX9.4*) were spread sparsely on chromosome 5, flanked by DNA transposons (i.e. *TcMart-Fot1*) or uncharacterized DNA repeats (Fig. 6B; also observed in *SIX8* as noted above). An additional copy of *SIX9.4* was identified on chromosome 12 of AT141, part of a 23-kb duplicated region between chromosome 5 and 12 (Fig. 5B). Based on the *SIX9* gene phylogeny (Supplementary Figure S7), sequences of FOLac *SIX9.1-9.4* were (nearly) identical to those in unrelated ff. spp., including *apii* races 3 and 4, *conglutinans*, *lini*, *coriandrii*,* niveum*, *semani*,* raphanin*, and *vasinfectum.*

#### SIX14

*SIX14* was consistently present among all the FOLac isolates, with no sequence variation among isolates, the exception being race 1 isolate Fol621 and race 1 variant VSP-0916 (Supplementary Figure S7), which had one non-synonymous mutation. *SIX14*, along with *SIX8* and *SIX9*, were all located on chromosome 5 of AT141 and at least 200-kb apart from one another (Fig. 5B), indicating that chromosome 5 is a putative pathogenicity chromosome. Likewise, in the genome of JCP043, we identified chromosome 15 as a putative pathogenicity chromosome because it harbored both *SIX9.4* and *SIX14* (Fig. 5A). Interestingly, the BLAST analysis revealed a second copy of *SIX14*, located on chromosome 10 in JCP043, however, it appeared to be disrupted by the insertion of a *Gypsy* retrotransposon (Fig. 6C), likely resulting in loss of function. This is consistent with a previous study, where a transposon had inserted into *SIX14* [43]. Compared to *SIX8* and *SIX9*,* SIX14* tended to have a limited distribution among the *F. oxysporum* taxa we examined as it was only identified in two unrelated ff. spp., including ff. spp. *niveum* and *lycopersici*. Sequences of *SIX14* identified among FOLac isolates were most closely related to those in f. sp. *niveum* (Supplementary Figure S7).

### Putative race-specific genes are clustered on several accessory chromosomes

We identified 1010 candidate unique genes for JCP043 (Fig. 4B), 563 of which were absent and 447 were partially present (below 90% identity or 80% coverage) in the AT141 genome. A total of 1081 candidate unique genes were identified in AT141, including 741 absent and 340 partially present in JCP043. After screening the candidate genes against nine representative FOLac genomes covering all four races, 687 putative race 1-specific genes and 536 putative race 4-specific genes were identified (Table 1 and Supplementary Files S6 and S7). Nearly 87% of the candidate race-specific genes were associated with the accessory genome regions and often clustered together (Supplementary Figure S5B). Chromosomes 7, 12 and 10 of JCP043 harbored the greatest numbers of race 1-specific genes (chr 7: 223, chr 12: 167, and chr10: 85). In AT141, chromosomes 13, 5, and 16 contained the most race 4-specific genes (chr13: 219, chr5: 76, and chr16: 60).

Among the FOLac race 1-specific genes, we identified 4 secreted CAZYmes, 14 effectors, 5 *mimp*-associated effectors, and 3 SM genes (Supplementary Table S5). As for the race 4-specific genes, 2 secreted CAZYmes, 8 effector, 3 *mimp*-associated effectors, and 6 SM genes were identified. Interestingly, two race-specific CAZYmes (JCP043_017764 and AT141_017966) were also predicted to be apoplastic effectors, potentially contributing to plant cell wall degradation. While most of the race-specific SM genes coded hypothetical proteins, we identified one unique SM gene cluster located on chromosome 16 in AT141 that harbored multiple race 4-specific genes, including a putative type-III polyketide synthase (AT141_019912) as the core biosynthetic enzyme, a glycoside hydrolase (GH) family protein (AT141_019910), a phosphate transporter (AT141_019913), and two hypothetical proteins (AT141_019917 and AT141_019918). The core biosynthetic enzyme had high similarity (88.5% identity in amino acid sequences) to the thiolase-like protein of *F. redolens* (Accession: XP_046053686.1).

### The T2T assembly reveals structural variants and putative genes not shown in contig-level assemblies

In comparison to the T2T assemblies of JCP043 and AT141, their respective Illumina assemblies presented several limitations in characterizing assembly and genomic features as described below and summarized in Table 1. The estimated genome sizes of JCP043 and AT141 based on their Illumina assemblies were 6.05 Mb and 14.92 Mb smaller, representing 83% and 77% of their T2T genome sizes, respectively. While the core genome regions were well represented in the Illumina assemblies of JCP043 and AT141, reflected in high BUSCO scores, only 48% and 46% of their accessory genome regions were recovered in their respective Illumina contigs, respectively. Since the Illumina assemblies were highly fragmented (5405 contigs for JCP043 and 5771 contigs for AT141), it was impossible to detect structural variants, including genome duplication and rearrangement, between the two isolates. Compared to the T2T assemblies, the Illumina assemblies of JCP043 and AT141 contained significantly fewer DNA repeats, representing only 6.46% and 4.86% of the genome sizes, respectively, in comparison to 18% and 20% in their respective T2T assemblies. Regarding the predicted genes, both Illumina assemblies turned out to miss ~ 10% of the total genes (1805 genes missing from JCP043 and 1972 missing from AT141), with 83% and 74% of them associated with ACs of JCP043 and AT141, respectively. Many of the missing genes were partially present due to the fact that they were located on the end of a contig. Illumina assemblies were able to capture the *SIX* genes and all but four *mimp* effectors, however, they fell short in identifying additional copies of *SIX8* and *SIX9.4*, as observed in all FOLac Illumina assemblies (Supplementary Table S4). When searching for putative race 1-specific genes in the Illumina assembly of JCP043, 171 were not detected, 150 of which were associated with ACs. The Illumina assembly of AT141 had 99 of the race 4-specific genes missing, with 88 of them located on ACs.

On the other hand, the NECAT assemblies of JCP043 and AT141, which were obtained by the NECAT assembler and error corrected using Illumina reads (without checking for misassembly and contig end extension), resulted in genome sizes very close to their corresponding T2T assemblies, as also observed for BUSCO scores (Table 1). The NECAT assemblies represented over 97% of both the core and accessory regions of each genome, with most of the NECAT contigs aligned with the T2T chromosomes (Supplementary Figure S9). However, the NECAT assemblies were less contiguous and did not have telomeres on many of the contigs, and produced a much smaller number of rDNA repeats (25 copies for JCP043 and 10 copies for AT141). The large-scale chromosome duplications identified from the T2T assemblies (i.e. the 1-Mb duplicated regions in JCP043 and the 500-kb inverted repeats in AT141) were not captured in the NECAT assemblies (Supplementary Figure S9). The NECAT assemblies were highly similar to the T2T assemblies regarding the amount and composition of repetitive elements, as well as predicted genes, with only ~ 100 genes absent. Not surprisingly, most of those missing genes were located within the large, duplicated regions. For instance, 92 out of 107 missing genes from the AT141 NECAT assembly were located on the 5’end of chromosome 17, which failed to be assembled in the NECAT assembly. The NECAT assemblies of both isolates possessed all the *mimp* effectors and rendered the same sequences and copy numbers for the *SIX* genes as the T2T assemblies. The NECAT assemblies possessed most of the race-specific genes, with only eight (for JCP043) and 23 (for AT141) genes missing compared to their respective T2T assemblies.

## Discussion

This study reports a complete (gapless and T2T complete) chromosome-level assembly for two key, very closely related *F. oxysporum* pathogens, which due to the highly repetitive nature of their extensive accessory genome regions, have heretofore evaded complete assembly. This result permitted comparative genomic analyses of the two T2T assemblies and revealed novel structural and genetic differences between the two races, which laid the groundwork to identify putative pathogenicity genes that may be associated with the host-pathogen interaction in the FOLac-lettuce pathosystem. More broadly, this study provides a workflow to generate T2T assemblies that can significantly improve our ability to investigate genome dynamics and organism adaptation, characterize genes that may be associated with pathogenicity, and identify race-specific sequences useful for diagnostics.

### A novel workflow for generating gapless, T2T assemblies and things to consider

In this study, we developed a novel workflow that deployed a suite of bioinformatic programs with various types of long and short sequencing technologies (ONT, Pacbio HiFi, Illumina, and Hi-C) to build *F. oxysporum* genome assemblies that are gapless and T2T complete. In summary, we considered the following three features as key players in achieving this goal. The first key feature relates to the abundance of long ONT reads (≥ 50 kb). These long reads are deemed crucial for T2T genome assembly because of their ability to resolve large repetitive regions in *F. oxysporum* accessory genome regions, some of which can reach as long as 20–30 kb in size. In our study, ONT reads that were above 50 kb provided 204× coverage for JCP043 and 176× coverage for AT141 (Table 1). To the best of our knowledge, such high long-read coverage has not been reported in any other *F. oxysporum* genome studies. The second key feature deals with misassembly, which is a major challenge in the assembly of highly repetitive accessory genome regions. Some of the misassembled regions failed to be detected by command-line programs because decent read coverage can be found at the misassembled regions but no reads entirely span them (Supplementary Figure S1). Our solution to this problem was to map long reads (≥ 50 kb) to the NECAT assembly using programs like CLC Genomics Workbench (QIAGEN, Aarhus, Denmark; https://digitalinsights.qiagen.com) and SeqMan NGen (DNASTAR, Madison, WI, USA), which enables visual scanning of the read mapping to identify misassembled regions. The third key feature involves the practice of read mapping-guided, iterative end extension on the intermediate contigs (see Methods for details). As far as we are aware of, there is no stand-alone bioinformatic software available to extend contigs using long reads beyond scaffolding and gap-filling capabilities. This is reflected in some of near complete *F. oxysporum* genomes (i.e. Fo47, Fo5176, and Fol4287) that have been published [48–50], none of which has achieved the level of genome completeness as the two FOLac assemblies presented here. An add-on value of using graphical read mapping for end extension is the ability to visually identify large-scale chromosome duplications due to read depth appearing doubled in the read mapping graphics, as observed in chromosomes 5 and 10 in JCP043 and chromosome 17 in AT141. These large, duplicated regions were not captured by the NECAT program (Supplementary Figure S9) or other long-read assemblers, including Flye [51] and Canu [52] that we tested. We are currently working on automating and fine-tuning the assembly pipeline, hoping to significantly reduce the amount of time needed to generate T2T assemblies.

Finally, the use of PacBio HiFi reads in conjunction with short read data for base-level error correction of the assembly is essential. The lack of Illumina read coverage in many regions of the T2T assembly (Supplementary Table S1) leaves sequence errors in those gapped regions unchecked. One contributing factor may be read depletion of highly repetitive regions early in the read mapping process. In contrast to short reads, HiFi reads exhibited uniformly high read coverage (> 100× coverage with reads ≥ 15 kb) across the entire genome (Supplementary Figures S2), thus allowing for gap-free error correction. Besides the heterogeneity in genome coverage, variability in Illumina read coverage was also found between different samples. A significantly higher proportion of the AT141 genome, including non-repetitive regions, was not covered by its own Illumina reads compared to that in JCP043, even though the total number of Illumina reads for AT141 was 30% greater than that for JCP043. These observations emphasize the importance of using read mapping to visually evaluate the uniformity of sequencing data used in genome polishing. While the PacBio HiFi data was important for error correction, due to read length it was not suitable as a standalone for *de novo* assemblies (for JCP043 the assembly yielded 185 contigs).

### T2T assemblies set a foundation for studying genome evolution in *F. oxysporum*

The T2T assemblies reported here revealed some novel structural differences in the accessory genome regions between FOLac race 1 and race 4, providing new insight regarding genome changes that may underlie the evolution of pathogenicity. The most distinct structural difference between them was that race 4 isolate AT141 possesses a genome 3.86 Mb larger than that of race 1 isolate JCP043 and organizes differently into 19 rather than 16 chromosomes (Table 1). Part of the larger genome size of AT141 was related to its inflated estimated copy number of rDNA repeats, which was 144 copies vs. 90 copies in JCP043. *F. oxysporum* f. sp. *radicis-cucumerinum* isolate Forc016 was estimated to have 98 rDNA copies [53], which was similar to that of JCP043. It is widely accepted that rDNA loci are dynamic and copy number fluctuates widely between individual within a species, and even likely between cells within a single organism [54]. Further analysis using quantitative real-time PCR is needed to determine the actual copy number of rDNA for the two isolates. Although similar difference in genome size between the two FOLac races was reported previously [43], the exact chromosome number difference between the two races was not resolved from those contig-level genomes. This has been the constraint in many comparative genomic analyses for members within *F. oxysporum.* An expended survey of additional FOLac races 1 and 4 isolates that have T2T assemblies available will help determine whether the genome size and number of ACs are fixed within a race or not.

Secondly, the T2T assemblies showed that repetitive elements made up 18–20% of the two FOLac genomes, which were substantially higher than those reported for other long-read assemblies, such as 10.54% for *F. oxysporum* f. sp. *cepae* [45], 15.4% for *F. oxysporum* f. sp. *conglutinans* and 16.42% for *F. oxysporum* f. sp. *lycopersici* [50]. Given that TEs play diverse roles in gene regulation, recombination, and adaptation to changing environment [55], the ability to identify an expanded pool of TEs from T2T assemblies serves as a valuable tool to understand how TEs contribute to pathogen diversification within *F. oxysporum*. Consistent with other studies [50, 56], common TEs, including DNA transposons and retroelements, were widely distributed among the accessory genome regions, with the exception of *Gypsy/DIRS1* elements, of which 75% were found in the core genome regions. The greater abundance of *Gypsy/DIRS1* elements in the core genome regions of *F. oxysporum* has also been reported in an opportunistic human pathogen, with nearly 90% of *Gypsy/DIRS1* elements found in the core genome [56]. While most of the repetitive elements were similar in copy number and length between the two isolates, a markedly larger number of uncharacterized repeats were associated with the race 4 genome (Supplementary Table S2), contributing to 44.8% (1.76 Mb in size) of the overall genome size difference between the two isolates. In particular, the accessory region on chromosome 8 in AT141 showed a very high level of uncharacterized repeats (Fig. 3) that was consistently found in the three published FOLac race 4 genomes (Supplementary Figure S4), suggesting that this putative proliferation may be a feature of FOLac race 4.

Thirdly, the T2T assemblies offered a unique opportunity to explore how large-scale chromosome duplications and rearrangements vary between the two FOLac races with the focus to identify genome regions that are race specific. Similar to the observations reported previously [43], the syntenic analysis between race 1 isolate JCP043 and race 4 isolate AT141 demonstrated highly conserved CCs but extremely fragmented and rearranged shared sequences among ACs (Figs. 2C and 3B), suggesting that each race underwent very different rearrangements resulting in loss of synteny. None of the ACs appeared to be exclusively associated with either of the two isolates, including chromosome 15 of AT141, which turned out to have many small non-repetitive sequences (2–6 kb in size) shared with JCP043. Our findings pointed to a previously unreported correlation between *Gypsy/DIRS1* elements and chromosomal translocation (Fig. 3A), which appeared to be a unique event in JCP043. In addition to their potential involvement in chromosomal translocation, *Gypsy/DIRS1* elements were found to be abundant near the boundaries of large, duplicated regions in JCP043 (a 1-Mb repeat duplicated between chromosomes 5 and 10) and AT141 (a 500-kb inverted repeat within chromosome 17) (Fig. 5), suggesting their additional role in large-scale chromosome duplications. The connection between *Gypsy/DIRS1* elements and chromosome rearrangements was recently reported in two stingless bee species, *Melipona quadrifasciata* and *M. scutellaris* [57], however, it remains an open question whether there is a causative relation between *Gypsy/DIRS1* elements and changes in genome architecture. A broader survey involving in-depth characterization of TEs among diverse *F. oxysporum* genomes representing different evolutionary lineages and ff. spp. will help better understand their role in *F. oxysporum* genome evolution. In addition to TEs, the enrichment of certain types of histone modifications or giant *Starship* elements, as described in *F. graminearum* [58] and *Macrophomna phaseolina* [59], respectively, may also contribute to the extensive genetic and large-scale genome variation in *F. oxysporum*.

### Plasticity of *SIX* genes in FOLac race 4 and their putative origin

With a wide collection of FOLac genomes representing all four races, the analysis of *SIX* genes showed the universal presence of *SIX9.4* and *SIX14* among all four races of FOLac. FOLac race 1 exhibits a unique *SIX* gene complement that distinguishes it from the three other races (Supplementary Table S4). Race 1 isolates do not have *SIX8* and three *SIX9* variants (*SIX9.1*, *SIX9.2*, and *SIX9.3*) while they are all present in races 2–4. However, in a recent study [42], PCR analysis of *SIX8*, *SIX9*, and *SIX14* in FOLac race 4 and race4 + isolates showed the absence of *SIX8* in many isolates, suggesting the instability of *SIX8* in the race 4 population. Besides, the race 4 isolates also showed high levels of intra-race variation in the three *SIX9* variants (*SIX9.1*, *SIX9.2*, and *SIX9.3*), with AP114 (originated from Denmark) harboring three of the variants while AL127 (originated from Ireland) having only *SIX9.2.* In addition, we found that AT141 was the only race 4 isolate that had two copies of *SIX8*, which was likely achieved through large-scale chromosome duplication (Fig. 5B) instead of a single gene duplication mediated by TEs. In contrast, we noticed stable presence of the *SIX* genes among all the race 1 isolates, with no sequence variation among isolates. Given the high level of plasticity in *SIX8* and *SIX9* within the race 4 population, which is likely driven by TEs that are present in close proximity to the *SIX* genes (Fig. 6), one should be cautious about using only *SIX* genes for discerning FOLac races as it may lead to erroneous results.

Interestingly, sequences of the three *SIX* genes present in FOLac were highly similar to those in ff. spp. that were closely related to FOLac race 3 (D. Geiser, unpublished). Moreover, the more ancestral FOLac race 2 lineage possessed all three *SIX* genes, including the four variants of *SIX9.* Given this information, we hypothesized either that these *SIX* genes may have existed in *F. oxysporum* before lineage diversification or that they have been transferred among different lineages of *F. oxysporum* after lineage diversification. Future study of the *SIX* gene complement with more extensive sampling of *F. oxysporum* genomes spanning all 17 lineages as delineated by D. Geiser (unpublished) will shed light on the evolution of the *SIX* gene family.

### Identification of putative race-specific genes provides a framework for functional characterization of candidate virulence/host-range genes

The race-specific genes that were identified in this study were validated against not only FOLac race 1 and 4 genomes, but also two representative isolates from FOLac races 2 and 3, which enabled more reliable identification of race-specific genes compared to previous studies. However, due to the lack of long-read assemblies for the race 2 and 3 isolates when this study was conducted, additional validation of race-specific genes against these two isolates was achieved through read mapping analysis to ensure accuracy, similar to the approach used to confirm *SIX9.2* was absent in AM163 (Supplementary Figure S8). Our results identified 687 putative race 1- and 536 putative race 4-specific genes, some of which encode putative secreted CAZymes, effectors (including *mimp*-associated effectors), and proteins involved in SM biosynthesis (Table 1 and Supplementary Table S5). Not surprisingly, nearly 87% of the race-specific genes were associated with ACs (Supplementary Figure S5B), with chromosomes 7 and 12 of JCP043 and chromosome 13 of AT141 each harboring over 100 race-specific genes, suggesting that they might be pathogenicity chromosomes. Chromosome 10 of JCP043 and chromosome 5 of AT141, which carried the *SIX* genes, also possessed many of the race-specific genes, raising the possibility of their roles in host range and virulence. Future studies are warranted for narrowing the candidates for race-specific genes, by screening them against a wide range of *F. oxysporum* ff. spp. and nonpathogenic isolates recovered from lettuce and other hosts. Also notable is the presence of various types of TEs within and/or in the flanking regions of the candidate race-specific genes. These observations raised the question of whether those genes are expressed *in planta*, due to potential disruptions in the promoter and coding sequences by transposon insertion, as observed previously in *SIX14* in FOLac race 1 isolate AJ520, which was found to be unexpressed during infection due to transposon insertion [43]. Transcriptome analyses of JCP043 and AT141 during lettuce infection are needed to help identify race-specific genes that are highly expressed *in planta* and to guide downstream functional characterization of candidate virulence genes to investigate their function in pathogenicity and host specificity.

### Is T2T genome assembly necessary?

While obtaining a gapless, T2T complete assembly would be ideal for genomic and genetic research, we found that NECAT assembly provides an accurate estimate of genome size, content and composition of DNA repeats, and gene content, including correct copy numbers for *SIX* genes (see Results for details). Moreover, generating NECAT assembly takes much less time than T2T assembly, which requires different types of bioinformatic tools and sequencing data to make it complete. Assembly using the NECAT program requires only one run of assembling ONT reads into contigs, followed by PacBio/Illumina-based error correction. Therefore, having a NECAT assembly may be an adequate solution for researchers interested in projects including pan-genome and basic repetitive sequence analyses. However, studies that aimed for identifying unique genes that may be associated with host specificity or detecting structural variants between individuals cannot be completed adequately without a T2T assembly. Short-read assembly, which has become a routine task for many research programs used in various downstream analyses, presents several additional limitations, including an incomplete set of predicted genes and inaccurate gene copy numbers, especially for those in ACs (see Results for details). Here we also cite cases where genes present in an assembly turned out to be absent based on read mapping (Supplementary Figure S8), and also where sequences thought to be absent turned out to be present. The latter case can be explained by the fact that parts of those genes land on the end of different contigs. Therefore, it is recommended that a combination of BLAST and read mapping analyses be used for accurate gene prediction, if long-read assembly is inaccessible.

## Conclusions

We present an assembly workflow that led to gapless, T2T complete genome assemblies for FOLac races 1 and 4, two devastating soilborne pathogens that have become increasingly prevalent in lettuce production areas worldwide. Comparative genomic analyses between the two isolates revealed major structural differences in the accessory genome regions and the potential involvement of *Gypsy/DIRS1* elements in chromosome duplication and translocation. We identified many putative race-specific genes that were uniquely present in one race while absent in three other races and warrant further investigation through transcriptome analyses during lettuce infection to understand their role in pathogenicity and host specificity. A comprehensive genomics study of multiple isolates representing all four races, along with a broad-spectrum phenotyping study to evaluate their host range and virulence, will allow us to reconstruct the evolutionary paths that led to host-specificity of *F. oxysporum* towards lettuce and subsequent diversification. Ultimately, the information we gained from the genomics research will greatly advance the development of effective management strategies to control Fusarium wilts.

### Methods

#### Culture growth

Fresh fungal mycelia used for long-read sequencing were prepared as follows. FOLac race 1 isolate JCP043 and race 4 isolate AT141 were started on potato dextrose agar (PDA; Difco Laboratories, Detroit, MI, USA) under dark incubation for seven days at 25 °C. Six agar plugs from colony edges were transferred to two Petri plates containing 30 ml of potato dextrose broth (PDB; Difco Laboratories) and incubated unagitated for two days at 25 °C. The resulting mother culture was blended with a sterile Waring laboratory blender (Conair LLC, Stamford, CT, USA) for 10 s to make a slurry, which was then mixed with 1.2 L of PDB and distributed to 60 Petri plates for large-scale liquid culture. The plates were incubated unagitated for two days at 25 °C. The resulting mycelia were harvested by centrifugation at 8,000 g for 15 min, washed twice with sterile distilled water, blotted dry, and flash frozen in liquid nitrogen before stored at -80 °C.

### High molecular weight (HMW) DNA extraction

Extraction of HMW DNA performed at the Michigan State University RTSF Genomics Core used a protocol adapted from the QIAGEN Genomic-tip protocol (QIAGEN, Germantown, MD, USA) while HMW DNA at the UC Davis DNA Technologies Core used a cetyl trimethyl ammonium bromide (CTAB)-based extraction protocol [60]. DNA quantity and purity were determined with the Qubit 4.0 fluorometer (Thermo Fisher Scientific) and the Nanodrop UV-Vis 45 spectrophotometer (Thermo Fisher Scientific, Wilmington, NC, USA), respectively. Integrity assessment of the genomic DNA was performed using a Femto Pulse system (Agilent Technologies, Santa Clara, CA, USA) and the Agilent 4200 TapeStation (Agilent Technologies). Short DNA fragments (≤ 25 kb) for the PacBio Revio library were eliminated using Blue Pippin (Sage Sciences, 50 Beverly, MA, USA). DNA samples with Nanodrop ratios 260/280 between 1.8 and 2.0, 260/230 between 2.0 and 2.2 and molecular weight ≥ 50 kb were selected for sequencing.

### Long-read whole genome sequencing

Long-read genome sequencing was carried out using HMW DNA on ONT PromethION and Pacific Biosciences HiFi platforms. The ONT sequencing was performed separately by two sequencing facilities, Michigan State University (for JCP043) and UC Davis (for AT141). Library for ONT sequencing was made using the Oxford Nanopore SQK-LSK114 Ligation Sequencing Kit V14 and run on a PromethION R10.4.1 flow cell. Sequencing was performed following manufacturer’s recommendations. MinKNOW software v.22.10.7 was used for data acquisition and base calling was achieved using Guppy v.6.3.9. Total yields for JCP043 and AT141 were 122Gb (7 million reads; read length N50 of 32 kb) and 149Gb (11 million reads; read length N50 of 21 kb), respectively. Raw reads were filtered with a Q score ≥ 9.0 and a minimum length of 10k, resulting in a total of 5.45 million reads for JCP043 and 9.45 million reads for AT141. HiFi reads were generated by the UC Davis DNA Technologies Core, with sequencing of JCP043 performed on the Sequel II system while AT141 on the Revio system. Total yields for JCP043 and AT141 were 12.5 Gb (1.1 M reads; read length N50 of 12 kb) and 62.7 Gb (5.9 M reads; read length N50 of 13 kb), respectively.

### T2T assembly

As summarized in Fig. 1, we developed a bioinformatic workflow that implemented a series of command-line and graphic user interface programs to assemble the two FOLac genomes, which is described in greater details in Supplementary File S1. Briefly, the Nanopore data assembler, NECAT version 0.0.1_update20200803 [61] was used to assemble ONT reads into a preliminary genome assembly, which was then visually inspected for mis-assembly by mapping 50–99 kb ONT reads to the contigs using CLC Genomics Workbench 22.0 (QIAGEN, Aarhus, Denmark; https://digitalinsights.qiagen.com/) and SeqMan NGen 16.0 (DNASTAR, Madison, WI, USA); due to the presence of repetitive sequences sometimes greater than 40 kb, reads below 50 kb were not used in mapping to reduce potential mis-assembly problems. Each program uses a different approach for read mapping that complements each other. CLC Genomics uses a percent identity and fraction overlap approach while SeqMan NGen uses a *k-*mer approach. The mis-assembled contigs were split into sub-contigs at the breakpoints before subject to a second run of read mapping, followed by end extension using the “Extend contig ends” function of the Genome Finishing Module on CLC Genomics. Other contigs, which did not have misassembled regions but had no telomeric repeats on either or both ends, were also included in the end extension analysis. After this procedure was repeated multiple times, the extended contigs and sub-contigs were merged where there was a minimum of 50-kb overlap using the nucmer module of the MUMmer package version 3.23 [62]. Once the T2T assembly was obtained, the continuity and correctness of the assembly was checked by re-mapping 50–99 kb ONT reads to the assembly using CLC Genomics, followed by repeating the read mapping using SeqMan NGen with a stringency setting of 95% and *k*-mer size of 30. The final T2T assembly was polished with two runs of HiFi reads mapping, followed by two runs of Illumina HiSeq reads mapping using SeqMan NGen with a stringency of 99% and *k*-mer size of 30. The NECAT contigs that contained the mt sequences were removed from the assembly, BLASTed against a reference mt genome of Fo47 (GenBank: LT906306.1) to identify a complete single-copy genome, and error corrected using Illumina data.

### Hi-C analysis

To verify that the T2T assembly of JCP043 is correct with no mis-assembled contigs, a proximity ligation-based method called, Hi-C analysis [63], was performed via Dovetail Omni-C library construction and sequencing on an Illumina HiSeqX platform by Cantata Bio. BWA-MEM version 0.7.17 [64] was used to align the Omni-C reads to the T2T assembly of JCP043, followed by Hi-C analysis using the SALSA2 pipeline [65]. The resulting alignment file was converted to create a .hic file and then visualized with JuiceBox [66].

### Genome assessment

QUAST version 5.3.0 [67] was used to assess the quality of the T2T assembly and to evaluate the genome completeness of the Illumina and NECAT assemblies with flags --eukaryote --fungus to specify a fungal organism, and --features set to gene to analyze gene content. Gene content of the assembly was determined based on the presence of BUSCOs using BUSCO version 5.4.5 [68], with the hypocreales_odb10 database.

### Analysis of the repeat content of the genome

We identified and classified repetitive and low complexity regions in the genome using RepeatModeler version 2.0.4 (http://www.repeatmasker.org). Then, the repeat library was used to analyze repetitive regions, as well as soft-masking the genome using RepeatMasker version 4.1.5 (https://www.repeatmasker.org/).

### Whole-genome comparison to identify core and accessory chromosomes

Core and accessory genome regions and chromosomes of JCP043 and AT141 were identified based on whole-genome alignment with the Fol4287 reference genome (GCA_000149955.2) using the nucmer module of the MUMmer package with the parameter –max-match, -L 10,000. The synteny plot was generated using TBtools-II v. 2.311 [69]. Chromosomes 5 and 14 in JCP043 and chromosome 8 in AT141 possessed large (> 0.5 Mb) accessory regions at one terminus; the boundaries of these regions were estimates based on MUMmer analysis for chromosome alignment between JCP043/AT141 and corresponding core regions of *F. oxysporum* f. sp. *lycopersici* isolate 4287 assembly (Supplementary File S8).

### Gene prediction and functional annotation

After repeat masking, we annotated the genome using Funannotate version 1.8.16 [70]. The masked genome file along with the RNA-seq data generated from mycelia of JCP043 grown under nine different environmental conditions (see below) were used as inputs to Funannotate to train the gene prediction models, followed by funannotate predict and funannotate update commands to annotate untranslated regions (UTRs) and refine gene model predictions. InterProScan5 version 5.64-96.0 [71] was used to assign functional annotation to predicted genes. Diamond blastp [72] was used to search UniProt DB v. 2023_04 [73] and MERPOP v. 12.0 [74] databases to aid in functional annotation and eggNOG terms were identified using eggNOG-mapper v. 2.1.12 [75]. Pfam domains were identified using PFAM v. 36.0 [76], and CAZymes were annotated using dbCAN v.12.0 [77]. Putative secreted proteins (secretomes) were identified through prediction of signal peptides using SignalP v.4.1 [78] and removing those predicted to contain transmembrane domains using the DeepTMHMM v.1.0 web server [79]. The resulting secretomes were used to predict effector proteins using EffectorP 3.0 [80]. Secondary metabolite gene clusters were identified using the antiSMASH fungal v. 8.0 web server [81] with the detection strictness set to relaxed. Prior to the antiSMASH analysis, the genome annotation file was filtered using AGAT v.1.4.3 [82] to retain only the longest isoform per gene. Genomic features, including gene density, distribution of TEs, and genome duplication events were visualized using TBtools-II v. 2.311. To conduct functional enrichment analysis, we used GOATOOLS v1.5.1 [83]. GO data v.1.2 (release date: 2025-07-22) was obtained from the Gene Ontology consortia [84, 85]. To avoid false positives, *p*-values were multi-test corrected using the Bonferroni method; the resulting adjusted *p*-values were subject to a significance threshold of 0.01.

### Identification of putative race-specific genes

To identify putative race-specific genes for FOLac races 1 and 4, we identified unique genes in JCP043 and AT141 first. CDS transcripts of the predicted genes were clustered to create a non-redundant gene set using the CD-HIT program version 4.8.1 [86]. Based on the criteria that were previously applied in similar analyses [45], the analysis was carried out with minimum 90% identity and minimum 80% coverage as the thresholds for clustering genes (cd-hit -c 0.9 -s 0.8). Pairs of homologous genes between the two genomes were then identified from the non-redundant gene sets using the RBBH module of the MMseq2 software version 18.8cc5c [87] with the parameters mmseqs easy-rbh --search-type 3 --min-seq-id 0.90 -c 0.8 --cov-mode 0. The synteny of the homologous genes in the core and accessory chromosomes was visualized using TBtools-II v. 2.311. The resulting non-homologous genes in JCP043 were extracted and BLASTed against the AT141 assembly using Geneious Prime version 24.0.7 (໿http://www.geneious.com/). The genes that were either absent in AT141 or partially present (below 90% identity or 80% coverage) were considered unique genes in JCP043. To validate their uniqueness to JCP043, raw Illumina reads of AT141 (SRR28734917) were aligned to the unique genes using SeqMan NGen with a stringency of 90% sequence identity and *k*-mer size of 21. Read coverage of the alignments was visually inspected with SeqMan Pro. Similarly, the unique genes in AT141 were identified following the same procedure and validated with read mapping analysis using raw Illumina reads of JCP043 (SRR28734937).

To further narrow down the unique genes that may be race-specific, the presence/absence analysis of the unique genes, including *mimp*-associated effectors (see below), for FOLac race 1 was carried out on four representative FOLac race 1 isolates (AJ520, AJ718, AJ865, and AT142), one isolate each for race 2 (F9501) and race 3 (FLK1001), and three FOLac race 4 isolates (AJ516, AJ592, AJ705), most of which had long-read assemblies. Details of the isolates can be found in Supplementary Table S4. BLAST search was conducted by querying the unique genes to each genome assembly via Megablast (Max E-value=1e-20, Match Mismatch = 1, -2, Gap Cost=linear) using Geneious Prime. A hit was considered present if the sequence identity and coverage were above 95%. The unique genes were also evaluated by read mapping analysis using SeqMan NGen for additional validation.

### Identification of *mimp*-associated effector proteins

To identify putative effectors associated with *mimps* for each FOLac genome, we employed the FoEC2 pipeline [47], which was designed specifically for identification of *mimp* effectors, with the parameters -g < genome_folder > and -a < annotation_folder>. Sequences of the resulting candidate effector protein set between 30 aa and 300 aa were then extracted from each genome and clustered using CD-HIT version 4.8.1 to create a non-redundant candidate effector set. To determine the presence/absence of JCP043 candidate effectors in AT141, initially, TBLASTN search using protein sequences of the candidate *mimp* effectors against the genome was performed, however the results seemed inaccurate due to introns that resulted in fragmented or incomplete hits. Instead, genomic DNA sequences of the non-redundant candidate *mimp* effectors of JCP043 were used to query against the genome of AT141 using Megablast on Geneious Prime, with an e-value cut-off of 1e^− 20^ and a percentage identity and coverage threshold of 90% and 80%, respectively. To identify candidate *mimp* effectors that are race 1 specific, we included nine additional FOLac isolates (the same set used in race-specific gene analysis as described above) in this analysis. Similarly, the presence/absence of AT141 candidate effectors in JCP043 and putative race 4-specific *mimp* effectors were identified following the same procedure.

### In silico assessment of *SIX* genes

To identify the *SIX* genes present in the *F. oxysporum* isolates used in this study (Supplementary Table S4), BLAST search was conducted by querying the *SIX* genes obtained from NCBI (*SIX1*: MK906592.1; *SIX2*: MK906595.1; *SIX3*: MK906598.1; *SIX4*:GQ268951.1; *SIX5*: MK906607.1; *SIX6*: MK906615.1; *SIX7*:GQ268954.1; *SIX8*: FJ755837.1; *SIX9*: KC701447.1; *SIX10*: MK906667.1; *SIX11*: MK906677.1; *SIX12*: MW160867.1; *SIX13*: MK906693.1; *SIX14*: KC701452.1) to each genome assembly via Discontiguous Megablast (Max E-value = 0.05, Match Mismatch = 2, -3, Gap Cost = 5, 2) using Geneious Prime. As for *SIX9*, sequences corresponding to the four variants of *SIX9* (*SIX9.1- SIX9.4*), which were reported in FOLac race 4 [43], were used as additional references to account for sequence diversity. A hit is valid if the query identity and coverage meet the 65% threshold. If the hit was similar to the reference (≥ 65% identity) but was low in coverage, Illumina reads of the target isolate were mapped to the corresponding *SIX* reference to retrieve full-length sequence using SeqMan NGen with stringency cutoffs of 90% sequence identity and *k*-mer size of 30. Sequences of the *SIX* genes were aligned using MUSCLE 5.1 [88] and then used as input for IQ-TREE v2.2.2.6 [89] with parameters ' -m MFP -B 1000 ' to create a phylogeny. The resulting maximum likelihood tree was visualized in FigTree, version 1.4.4 (http://tree.bio.ed.ac.uk/software/figtree/). Sequences of *SIX8*, *SIX9*, and *SIX14* identified from all the *F. oxysporum* isolates used in this study are provided in Supplementary Files S3-S5.

### RNA extraction and sequencing

For *F. oxysporum* genome annotation, RNA-seq data obtained from PDB-cultured mycelia has been commonly used as the standard to train the gene prediction models. Considering that environmental factors (i.e. pH, nutrient composition, temperature and light) have significant impact on fungal gene expression [90, 91], we generated nine RNA-seq libraries from race 1 isolate JCP043 that was grown under different growth conditions (see below) to expand the expression of a wide variety of genes. Six agar plugs of five-day old JCP043 culture were inoculated in 30 ml PDB at 25 °C for two days. The mycelia were washed twice with sterile water before blended with water. Ten milliliters of the mycelial slurry were transferred to each bottle containing 100 ml of a specific type of media listed below, mixed well, and then distributed to four petri plates to grow for 48 h at 25 °C in the darkness. The media included PDB (pH of 6), PDB with 4% NaCl, PDB adjusted to a pH of 9 with NaOH, PDB amended with 0.5% and 1% peptone, respectively, carboxymethyl cellulose (CMC), and filter-sterilized macerated lettuce crown extract, prepared according to the method described previously [92]. Additional growth conditions include mycelia grown in PDB for 20 h at 25 °C before switching to 37 °C for 4 h, and mycelia grown in PDB under 24-h light. Mycelia were collected from the nine growth conditions by removing liquid from the tissue using filtration, washing the tissue with sterile water twice, and blotting the tissue dry. The tissue mat was then partitioned into 2 ml Eppendorf tubes, each containing approximately 100 mg of wet tissue, and immediately flash frozen in liquid nitrogen. The frozen tissue was stored at -80 C until the RNA was extracted.

RNA was extracted using TRIzol reagent (Life Technology, Karlsruhe, Germany) and purified with a Zymo RNA clean and Concentrate kit (Zymo, Irvine, CA, USA), according to the manufacturer’s instructions. The quality and quantity of purified RNA were determined using the NanoDrop spectrophotometer and Agilent 4200 TapeStation. All the RNA samples, which yielded RIN scores above 9, were used for sequencing. Libraries were prepared at the Michigan State University RTSF Genomics Core using the Watchmaker Genomics mRNA Library Preparation kit (Watchmaker Genomics, Boulder, CO) with IDT xGEN 10nt Unique Dual-Index primers (Integrated DNA Technologies, Coralville, IA) following manufacturer’s recommendations. Completed libraries were assessed for quality and quantified using a combination of Biotium AccuGreen High Sensitivity dsDNA (Biotium, Frement, CA, USA) and Agilent 4200 TapeStation. All libraries were barcoded, normalized, and pooled equimolarly for sequencing in an AVITI Cloudbreak Freestyle High Output flow cell in a 2 × 150 bp paired end format. Base calling was done by AVITIOS v3.2.0 and the output was demultiplexed and converted to FastQ format using Element Biosciences bases2fastq v2.1.0. Approximately 55–70 million high-quality (% ≥ Q30 reads was ∼90%) paired-end reads were obtained for each library with a total yield of 183.5 Gb of sequencing data.

## Supplementary Information

Supplementary Material 1: Step-by-step instructions for generating T2T assembly.

Supplementary Material 2: DNA sequences of uncharacterized repeats in JCP043 and AT141. DNA sequences of SIX8 from 27 F. oxysporum isolates used in this study. DNA sequences of SIX9 (SIX9.1, SIX9.2, SIX9.3 and SIX9.4) from 72 F. oxysporum isolates used in this study. DNA sequences of SIX14 from 61 F. oxysporum isolates used in this study. DNA sequences of race 1-specific genes. DNA sequences of race 4-specific genes. MUMer analysis for chromosome alignment between JCP043/AT141 and Fol4287 showing the boundaries between core and accessory regions of the chromosome.

Supplementary Material 3: Table S1. Gaps in coverage for the T2T assemblies of JCP043 (left) and AT141 (right) when mapping their respective Illumina reads. Table S2. Categories and abundance of repetitive DNA in JCP043 and AT141. Table S3. GO terms enriched in the accessory genome regions of JCP043 and AT141. Table S4. Long-read and short-read assemblies used in the analysis of SIX genes and race-specific genes. Table S5. Detailed information of several specific gene classes that are race 1 and race 4 specific based on the analysis of nine FOLac genomes. Figure S1. ONT reads mapping to the NECAT assembly of AT141 before and after correction was made on the misassembled region. (A) Misassembled region marked in blue box was identified based on a sharp decrease in read depth with a breakpoint marked with an arrow. Mismatches in reads are indicated with light colors. Correction was made by splitting the contig into two pieces at the breakpoint, followed by end extension and rejoining. (B) The updated version of the contig was confirmed to be accurate based on uniform and continuous read coverage. Reads coded in red and green indicate forward and reverse directions, respectively. Figure S2. Comparison of Illumina (top panel) vs. PacBio HiFi reads (bottom panel) mapping to the same accessory region of JCP043. Illumina reads displayed low and zero read coverage in multiple regions, whereas continuous and uniform read coverage was observed using PacBio HiFi reads. Figure S3. Genome-wide Hi-C contact map showing the interaction matrices among 16 chromosomes. The bright red diagonal band within each square represents elevated contact frequency along the chromosome, indicating correct genome organization. Figure S4. Dot plots depicting the synteny between the T2T assemblies for race 1 and 4 isolates reported herein and each of the six published FOLac race 1 and 4 assemblies (Bastes et al. 2024). The nucmer package (nucmer –maxmatch; -L 10000) was used to identify highly similar regions, followed by data visualization with Assemblytics (Nattestad and Schatz 2016). (A) Race 1 JCP043 (Reference) accessory chromosomes 7, 10, 12 and 15 showed great synteny with the three FOLac race 1 isolates but not with the three FOLac race 4 isolates, which are highlighted in blue dotted rectangular boxes. (B) Race 4 AT141 (Reference) accessory chromosomes 5, 12, 13, 15, 17, and 19 showed great synteny with the three FOLac race 4 isolates but not with the three FOLac race 1 isolates, which are highlighted in blue dotted rectangular boxes. Figure S5. Visualization of homologous gene pairs and the distribution of virulence and race-specific genes in the (A) core and (B) accessory genomes of JCP043 and AT141. Order of tracks from inward to outward (a-e) represent the location of: a: secondary metabolite (SM) gene clusters, b: secreted carbohydrate-activate enzymes (CAZYmes), c: effector genes, d: mimp-associated effector genes, and e: race-specific genes. Gene IDs of the race-specific virulence genes, including SM genes (blue), secreted CAZYmes (black), effectors (pink), and mimp-associated effectors (green), are shown outside of track e. See Supplementary Table S5 for detailed information of the abovementioned race-specific genes. Lines in the center represent pairs of homologous genes (15063 pairs in the core; 917 pairs in the accessory) identified with reciprocal best BLAST hit. Figure S6. MUSCLE alignment of SIX8 sequences from F. oxysporum ff. spp. conglutinans, lactucae, lycopersici, and niveum. Nucleotide with 100% identity among all the sequences are indicated with grey bars, whereas gaps and indels are indicated with horizontal lines. Single nucleotide polymorphisms are noted with colored lines. Identity scale for the consensus sequence shown on the top: green=100%, gold=30-99.9%. Figure S7. Phylogenetic analysis of SIX8, SIX9, and SIX14 using 87 F. oxysporum genomes. Maximum likelihood trees of (A) SIX8, (B) SIX9, and (C) SIX14, inferred using IQ-TREE 2 [89]. Each tree is rooted through the F. oxysporum f. sp. lycopersici SIX gene references. The numbers above the branches indicate bootstrap values. Taxon names of FOLac isolates are highlighted in blue. Figure S8. Coverage of Illumina reads from AM163 (FOLac race 1) mapping to the reference sequences of SIX9.2, SIX9.4, and SIX14 using SeqMan NGen. Mapped reads that were one-directional and both-directional were indicated in blue and green lines, respectively. Red lines indicate regions where only one read was mapped. Detailed description of the coverage graph is shown in the box inserted in SIX9.2 read mapping graph. Coverage threshold is set to 2×. Figure S9. Dot plots depicting the synteny between the T2T and NECAT assemblies of (A) JCP043 and (B) AT141. The nucmer package (nucmer –maxmatch; –L 10000) was used to identify highly similar regions, followed by data visualization with Assemblytics (Nattestad and Schatz 2016).

## Data Availability

The datasets generated for this study can be found in the article and Supplementary Material. All raw sequencing data have been submitted to the NCBI under the BioProject ID PRJNA1098703 with the accession numbers of SRR35856514- SRR35856527. The final assembled genomes are deposited under the same BioProject at NCBI. (https://figshare.com/s/60447a577ab459285236). Further inquiries can be directed to the corresponding author.

## Abbreviations

CC: Core chromosome
AC: Accessory chromosome
FOLac: F. oxysporum f. sp. Lactucae
T2T: Telomere-to-telomere
SIX: Secreted in Xylem
ff. spp.: Formae speciales
f. sp.: Forma specialis
TE: Transposable element
ONT: Oxford Nanopore Technologies
mt: Mitochondrial
rDNA: Ribosomal deoxyribonucleic acid
SNP: Single nucleotide polymorphism
BUSCO: Benchmarking universal single-copy ortholog
BLAST: Basic local alignment search tool
LTR: Long terminal repeat
LINE: Long interspersed nuclear element
CAZYme: Carbohydrate-active enzyme
SM: Secondary metabolite
GO: Gene ontology
mimp: Miniature impala
GH: Glycoside hydrolase
PDA: Potato dextrose agar
PDB: Potato dextrose broth
HMW: High molecular weight
CTAB: Cetyl trimethyl ammonium bromide
Hi-C: High-throughput chromosome conformation capture
UTR: Untranslated region
CDS: Coding sequence
RNA: Ribonucleic acid
CMC: Carboxymethyl cellulose
aa: Amino acid
