# Supplementary material for: Assembling telomere-to-telomere genomes of Fusarium oxysporum f. sp. lactucae provides a roadmap for studying genome and phenotype evolution
Source: BMC Genomics. 2026 Apr 7;27:406. doi: 10.1186/s12864-026-12744-5 (PMC13104227; doi:10.1186/s12864-026-12744-5)
Supplement: Supplementary file 1 — Supplementary Material 1: Step-by-step instructions for generating T2T assembly. [file 12864_2026_12744_MOESM1_ESM.docx]

**Supplementary File S1**

**Step-by-step instructions for generating T2T assembly**

**Step 1 – NECAT assembly:** The Nanopore data assembler, NECAT version 0.0.1_update20200803 (Chen et al. 2021) was used as an error correction and de novo assembly tool for generating the preliminary genome assembly by defining the following parameters in the program’s configuration file: genome_size=63Mb, min_read_length=10k, prep_output_coverage=40, cns_output_coverage=30. The remaining parameters were kept as default. The contigs, which typically span between 100-150 kb in size, that contained the mitochondrial DNA sequences were identified by BLAST against the complete mt genome of Fo47 (GenBank: LT906306.1). The NECAT assembly after removing the contigs representing the mt genome was carried forward for the downstream analyses.

**Step2 – Mis-assembly inspection:** The NECAT assembly was inspected for mis-assembled regions, which often occurred in repeat-rich regions of the genome, by mapping 50-99 kb ONT reads to the contigs using the “Map Reads to Reference” module on CLC Genomics Workbench version 22 (QIAGEN, Aarhus, Denmark; https://digitalinsights.qiagen.com/) with the following parameters: Match score: 1, Mismatch cost: 2, Linear gap cost, Similarity fraction: 0.96, Length fraction:0.7. A semi-automated search for mis-assembled region in the read mapping graphics was achieved using the “Find Low Coverage” function with a custom threshold of 50. Once the mis-assembled regions have been identified for one contig, the new consensus sequence was generated based on the read mapping with the following options set to: low coverage definition threshold=50, remove regions with low coverage, split into separate sequences. By doing this, some of the original contigs were split into multiple sub-contigs, eliminating the mis-assembled regions. For the contigs free of mis-assembly, the new consensus sequence was exported. Alternatively, SeqMan NGen 16.0 (DNASTAR, Madison, WI, USA) can be used to check for misassembly with a stringency setting of 95% and *k*-mer size of 30.

**Step 3 – End extension:** The contigs and sub-contigs generated from Step 2 were subject to a second run of read mapping using the same settings as described above, followed by end extension using the “Extend contig ends” function of the Genome Finishing Module on CLC Genomics Workbench (Minimum coverage: 3, Maximum unaligned ends coverage: 30%), which typically extended the contig by 15-30 kb on both ends. The process for end extension was performed iteratively until the end contained telomeric repeats or rDNA sequences. In a few cases, HiFi reads (≥ 15 kb) were employed to extend certain contigs that display low read coverage on the ends. We also ran into situations where no consensus sequence could be called on the extended region because of the heterogeneity of the mapped reads. This often occurred in repetitive regions where many of the aligned reads may belong somewhere else. Manual extension was conducted by extracting all the reads aligned to the end of the contig, imported them to Geneious Prime version 24.0.7 (﻿<http://www.geneious.com/>) for alignment, identified the longest read, and attached it to the end of the consensus sequence. The resulting extended contig was then used as input sequence to continue the end extension.

In the processing of end extension, we encountered one particularly interesting contig in AT141, which had the 5’end fully extended to reach the telomere while the 3’end failed to extend any further. It was speculated that the 3’end might be part of an inverted repeat region that is reverse complimentary to the 5’end of the contig. This speculation was supported by two read mapping observations: a) the 3’end of the contig was 100% match to the 5’end of the contig; b) the 5’end had double the read coverage compared to the rest of the contig (~300× vs ~150×). To allow the end extension to continue at the 3’end, we masked the 5’end of the contig (~500 kb) prior to read mapping. After around ten rounds of progressive end extension towards the 3’end direction, the chromosome was brought to completeness with telomeric repeats on both ends.

**Step 4 – Contig merging**: The extended contigs and sub-contigs were merged when there was a minimum of 50-kb overlap using the nucmer module of the MUMmer package version 3.23 (Kurtz et al. 2004). Once the T2T assembly was obtained, the continuity and correctness of the assembly was checked by re-mapping 50-99 kb ONT reads to the assembly using using CLC Genomics with the same settings as described above, followed by repeating the read mapping using SeqMan NGen with a stringency setting of 95% and *k*-mer size of 30.

**Step 5 –Error correction:** SeqMan NGen was deployed in this procedure as it allowed for visual inspection of nucleotide variants and corresponding read coverage. The T2T assembly was polished with two types of high-accuracy sequencing data: Illumina HiSeq reads (150 bp, paired-end) generated from our previous study (Li et al. 2025) and HiFi reads (≥ 15 kb). Error correction was performed sequentially with two runs of HiFi reads mapping followed by two runs of HiSeq reads mapping using SeqMan NGen with a stringency of 99% and *k*-mer size of 30. Consensus sequences were called based on at least 75% of the mapped reads. Manual analysis of mapped reads using SeqMan Pro was performed to resolve the issue with degenerate bases.
