# Supplementary figures and images for: Assembling telomere-to-telomere genomes of Fusarium oxysporum f. sp. lactucae provides a roadmap for studying genome and phenotype evolution
Source: BMC Genomics. 2026 Apr 7;27:406. doi: 10.1186/s12864-026-12744-5 (PMC13104227; doi:10.1186/s12864-026-12744-5)

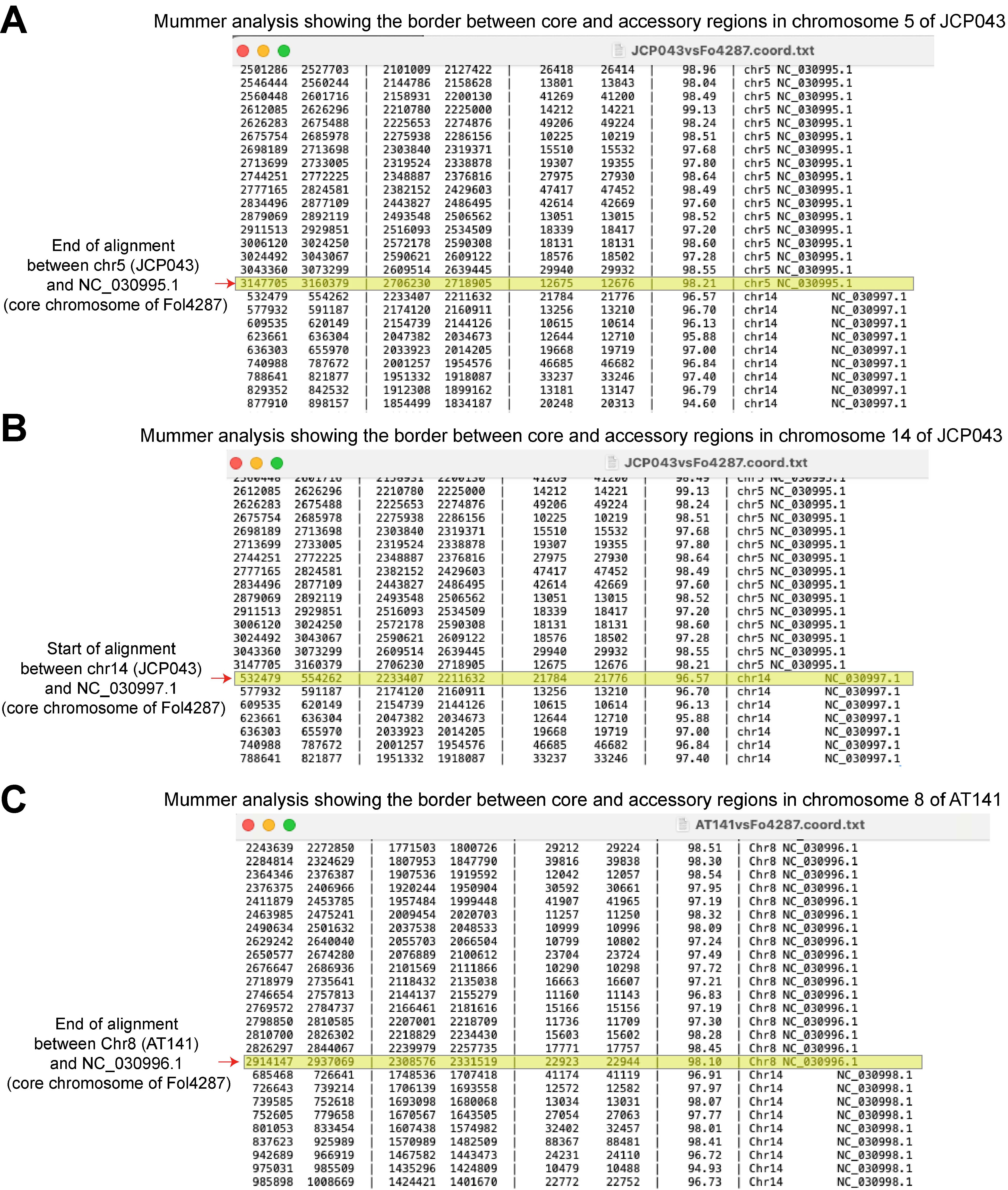

Supplement: Supplementary file 2 — Supplementary Material 2: DNA sequences of uncharacterized repeats in JCP043 and AT141. DNA sequences of SIX8 from 27 F. oxysporum isolates used in this study. DNA sequences of SIX9 (SIX9.1, SIX9.2, SIX9.3 and SIX9.4) from 72 F. oxysporum isolates used in this study. DNA sequences of SIX14 from 61 F. oxysporum isolates used in this study. DNA sequences of race 1-specific genes. DNA sequences of race 4-specific genes. MUMer analysis for chromosome alignment between JCP043/AT141 and Fol4287 showing the boundaries between core and accessory regions of the chromosome. [file 12864_2026_12744_MOESM2_ESM.zip › 12864_2026_12744_MOESM2_ESM-updated/Supplementary File S8.jpg]
