# Supplementary material for: Assembling telomere-to-telomere genomes of Fusarium oxysporum f. sp. lactucae provides a roadmap for studying genome and phenotype evolution
Source: BMC Genomics. 2026 Apr 7;27:406. doi: 10.1186/s12864-026-12744-5 (PMC13104227; doi:10.1186/s12864-026-12744-5)
Supplement: Supplementary file 3 — Supplementary Material 3: Table S1. Gaps in coverage for the T2T assemblies of JCP043 (left) and AT141 (right) when mapping their respective Illumina reads. Table S2. Categories and abundance of repetitive DNA in JCP043 and AT141. Table S3. GO terms enriched in the accessory genome regions of JCP043 and AT141. Table S4. Long-read and short-read assemblies used in the analysis of SIX genes and race-specific genes. Table S5. Detailed information of several specific gene classes that are race 1 and race 4 specific based on the analysis of nine FOLac genomes. Figure S1. ONT reads mapping to the NECAT assembly of AT141 before and after correction was made on the misassembled region. (A) Misassembled region marked in blue box was identified based on a sharp decrease in read depth with a breakpoint marked with an arrow. Mismatches in reads are indicated with light colors. Correction was made by splitting the contig into two pieces at the breakpoint, followed by end extension and rejoining. (B) The updated version of the contig was confirmed to be accurate based on uniform and continuous read coverage. Reads coded in red and green indicate forward and reverse directions, respectively. Figure S2. Comparison of Illumina (top panel) vs. PacBio HiFi reads (bottom panel) mapping to the same accessory region of JCP043. Illumina reads displayed low and zero read coverage in multiple regions, whereas continuous and uniform read coverage was observed using PacBio HiFi reads. Figure S3. Genome-wide Hi-C contact map showing the interaction matrices among 16 chromosomes. The bright red diagonal band within each square represents elevated contact frequency along the chromosome, indicating correct genome organization. Figure S4. Dot plots depicting the synteny between the T2T assemblies for race 1 and 4 isolates reported herein and each of the six published FOLac race 1 and 4 assemblies (Bastes et al. 2024). The nucmer package (nucmer –maxmatch; -L 10000) was used to identify highly simi [file 12864_2026_12744_MOESM3_ESM.docx]

Supplementary Table S1. Gaps in coverage for the T2T assemblies of JCP043 (left) and AT141 (right) when mapping their respective Illumina reads.

| **JCP043 (race 1)** | | | | **AT141 (race 4)** | | | |
| --- | --- | --- | --- | --- | --- | --- | --- |
| **Chr #^1^** | **Length (bp)** | **Sequencing gaps^2^ (bp)** | **Fraction**  **(%)** | **Chr #^1^** | **Length (bp)** | **Sequencing gaps^2^ (bp)** | **Fraction**  **(%)** |
| chr1 | 6,770,669 | 3,110 | 0.046 | Chr1 | 6,819,533 | 191,593 | 2.809 |
| chr2 | 6,250,436 | 4,438 | 0.071 | Chr2 | 5,908,551 | 62,362 | 1.055 |
| chr3 | 5,608,331 | 1,544 | 0.028 | Chr3 | 5,848,757 | 107,868 | 1.844 |
| chr4 | 5,459,532 | 366 | 0.007 | Chr4 | 5,717,665 | 167,139 | 2.923 |
| chr5-Core | 3,160,379^3^ | 115 | 0.003 | Chr6 | 4,695,532 | 109,298 | 2.328 |
| chr6 | 4,634,744 | 6,887 | 0.149 | Chr7 | 4,252,408 | 101,175 | 2.379 |
| chr8 | 4,236,707 | 4,633 | 0.109 | Chr8-Core | 2,937,069^3^ | 164,308 | 5.463 |
| chr9 | 3,809,017 | 26 | 0.001 | Chr9 | 3,799,817 | 134,667 | 3.544 |
| chr11 | 3,058,378 | 5,270 | 0.172 | Chr10 | 3,422,730 | 149,756 | 4.375 |
| chr13 | 2,775,815 | 4,905 | 0.177 | Chr11 | 2,930,467 | 183,868 | 6.274 |
| chr14-Core | 2,725,597^3^ | 25 | 0.001 | Chr14 | 2,651,588 | 101,455 | 3.826 |
| **chr5-AC** | **1,522,688^3^** | **16,376** | **1.19** | **Chr5** | **5,686,892** | **125,778** | **2.212** |
| **chr7** | **4,314,348** | **30,493** | **0.707** | **Chr8-AC** | **960,134^3^** | **20,492** | **2.303** |
| **chr10** | **3,246,481** | **33,412** | **1.029** | **Chr12** | **2,925,940** | **184,934** | **6.32** |
| **chr12** | **2,894,522** | **12,307** | **0.425** | **Chr13** | **2,740,747** | **37,891** | **1.383** |
| **chr14-AC** | **532,478^3^** | **0** | **0** | **Chr15** | **1,904,113** | **41,373** | **2.173** |
| **chr15** | **2,314,100** | **28,685** | **1.24** | **Chr16** | **1,746,802** | **185,063** | **10.594** |
| **chr16** | **1,322,771** | **4,183** | **0.316** | **Chr17** | **1,714,834** | **62,957** | **3.671** |
| Total | 64,636,993 | 156,775 | 0.243 | **Chr18** | **1,010,980** | **18,841** | **1.864** |
|  |  |  |  | **Chr19** | **827,607** | **22,511** | **2.72** |
|  |  |  |  | Total | 68,502,166 | 2,173,329 | 3.219 |

^1^Accessory (AC) chromosomes and genome regions are in bold.

^2^Illumina reads from JCP043 and AT141 were mapped to their corresponding T2T genome assembly with 99% sequence identity to ensure the mapping accuracy. Genomic regions where read depth is below 3× are considered sequencing gaps, and the sum of the gaps are shown per chromosome.

^3^Boundaries of the core and accessory regions of the chromosome were estimated by MUMmer analysis for chromosome alignment between JCP043/AT141 and corresponding core regions *F. oxysporum* f. sp. *lycopersici* isolate 4287 assembly.

Supplementary Table S2. Categories and abundance of repetitive DNA in JCP043 and AT141.

| Repeat class | JCP043 (race 1) | | |  | AT141 (race 4) | | |
| --- | --- | --- | --- | --- | --- | --- | --- |
|  | # of elements | Length (bp) | Percentage of sequence |  | # of elements | Length (bp) | Percentage of sequence |
| **Total** |  |  |  |  |  |  |  |
| **DNA transposons** |  |  |  |  |  |  |  |
| *hobo-Activator (hAT)* | 1,823 | 2,053,291 | 3.20% |  | 1,984 | 1,923,317 | 2.81% |
| *Tc1-IS630-Pogo* | 1,255 | 1,354,848 | 2.11% |  | 1,549 | 1,597,867 | 2.33% |
| *MULE-MuDR* | 532 | 599,642 | 0.94% |  | 789 | 804,886 | 1.17% |
| *PiggyBac* | 499 | 290,341 | 0.45% |  | 481 | 297,204 | 0.43% |
| *Helitron* | 107 | 241,750 | 0.38% |  | 125 | 214,530 | 0.31% |
| **LTR retrotransposons** | | | | | | | |
| *Gypsy/DIRS1* | 1,183 | 1,668,640 | 2.60% |  | 1,095 | 1,825,698 | 2.67% |
| *Ty1/Copia* | 521 | 621,320 | 0.97% |  | 672 | 622,497 | 0.91% |
| **LINE retrotransposons** | 797 | 908,800 | 1.42% |  | 939 | 1,147,814 | 1.68% |
| **Uncharacterized** | 7,831 | 2,612,862 | 4.08% |  | 9,908 | 4,339,046 | 6.33% |
| **Satellites** | 28 | 109,653 | 0.17% |  | 91 | 364,184 | 0.53% |
| **Simple repeats** | 6,839 | 284,043 | 0.44% |  | 7,007 | 292,105 | 0.43% |
| **Low complexity** | 774 | 36,439 | 0.06% |  | 777 | 36,996 | 0.05% |

Abbreviations: LTR: Long Terminal Repeat; LINE: Long Interspearsed Nuclear Elements.

Supplementary Table S3. GO terms enriched in the accessory genome regions of JCP043 and AT141.

| **JCP043** | | | **AT141** | | |
| --- | --- | --- | --- | --- | --- |
| **GO identifier^1^** | **GO category^2^** | **GO description** | **GO identifier^1^** | **GO category^2^** | **GO description** |
| GO:0043171 | BP | peptide catabolic process | **GO:0070212** | **BP** | **protein poly-ADP-ribosylation** |
| **GO:0048878** | **BP** | **chemical homeostasis** | GO:0006302 | BP | double-strand break repair |
| GO:0033540 | BP | fatty acid beta-oxidation using acyl-CoA oxidase | **GO:0030003** | **BP** | **intracellular monoatomic cation homeostasis** |
| **GO:0042592** | **BP** | **homeostatic process** | **GO:0006873** | **BP** | **intracellular monoatomic ion homeostasis** |
| GO:0006518 | BP | peptide metabolic process | **GO:0050801** | **BP** | **monoatomic ion homeostasis** |
| **GO:0006873** | **BP** | **intracellular monoatomic ion homeostasis** | **GO:0055082** | **BP** | **intracellular chemical homeostasis** |
| **GO:0030003** | **BP** | **intracellular monoatomic cation homeostasis** | **GO:0055080** | **BP** | **monoatomic cation homeostasis** |
| **GO:0055080** | **BP** | **monoatomic cation homeostasis** | **GO:0048878** | **BP** | **chemical homeostasis** |
| **GO:0055082** | **BP** | **intracellular chemical homeostasis** | **GO:0019725** | **BP** | **cellular homeostasis** |
| **GO:0050801** | **BP** | **monoatomic ion homeostasis** | **GO:0042592** | **BP** | **homeostatic process** |
| **GO:0051091** | **BP** | **positive regulation of DNA-binding transcription factor activity** | **GO:0031930** | **BP** | **mitochondria-nucleus signaling pathway** |
| **GO:0051090** | **BP** | **regulation of DNA-binding transcription factor activity** | **GO:0051452** | **BP** | **intracellular pH reduction** |
| GO:0055088 | BP | lipid homeostasis | **GO:0007035** | **BP** | **vacuolar acidification** |
| **GO:0051452** | **BP** | **intracellular pH reduction** | GO:0035337 | BP | fatty-acyl-CoA metabolic process |
| **GO:0007035** | **BP** | **vacuolar acidification** | **GO:0051090** | **BP** | **regulation of DNA-binding transcription factor activity** |
| GO:0009062 | BP | fatty acid catabolic process | **GO:0051091** | **BP** | **positive regulation of DNA-binding transcription factor activity** |
| **GO:0070212** | **BP** | **protein poly-ADP-ribosylation** | GO:0000724 | BP | double-strand break repair via homologous recombination |
| GO:0034440 | BP | lipid oxidation | **GO:0030641** | **BP** | **regulation of cellular pH** |
| **GO:0019725** | **BP** | **cellular homeostasis** | **GO:0006885** | **BP** | **regulation of pH** |
| GO:0072329 | BP | monocarboxylic acid catabolic process | **GO:0051453** | **BP** | **regulation of intracellular pH** |
| **GO:0051453** | **BP** | **regulation of intracellular pH** | GO:0035336 | BP | long-chain fatty-acyl-CoA metabolic process |
| **GO:0006885** | **BP** | **regulation of pH** | GO:0000725 | BP | recombinational repair |
| **GO:0030641** | **BP** | **regulation of cellular pH** | GO:0033559 | BP | unsaturated fatty acid metabolic process |
| GO:0009116 | BP | nucleoside metabolic process | GO:0098660 | BP | inorganic ion transmembrane transport |
| GO:0006635 | BP | fatty acid beta-oxidation | GO:0032107 | BP | regulation of response to nutrient levels |
| GO:0019395 | BP | fatty acid oxidation | GO:0006808 | BP | regulation of nitrogen utilization |
| GO:0030258 | BP | lipid modification | GO:0006812 | BP | monoatomic cation transport |
| GO:1901657 | BP | glycosyl compound metabolic process | GO:0001676 | BP | long-chain fatty acid metabolic process |
| **GO:0031930** | **BP** | **mitochondria-nucleus signaling pathway** | **GO:1990404** | **MF** | **NAD+-protein mono-ADP-ribosyltransferase activity** |
| GO:0015074 | BP | DNA integration | **GO:0003950** | **MF** | **NAD+ poly-ADP-ribosyltransferase activity** |
| GO:0009450 | BP | gamma-aminobutyric acid catabolic process | GO:0009378 | MF | four-way junction helicase activity |
| GO:0009448 | BP | gamma-aminobutyric acid metabolic process | GO:0043138 | MF | 3'-5' DNA helicase activity |
| GO:0016042 | BP | lipid catabolic process | **GO:0016705** | **MF** | **oxidoreductase activity, acting on paired donors, with incorporation or reduction of molecular oxygen** |
| GO:0003997 | MF | acyl-CoA oxidase activity | GO:0003678 | MF | DNA helicase activity |
| GO:0033293 | MF | monocarboxylic acid binding | GO:0046983 | MF | protein dimerization activity |
| GO:0005504 | MF | fatty acid binding | **GO:0005506** | **MF** | **iron ion binding** |
| GO:0016634 | MF | oxidoreductase activity, acting on the CH-CH group of donors, oxygen as acceptor | GO:0016763 | MF | pentosyltransferase activity |
| GO:0043177 | MF | organic acid binding | **GO:0004497** | **MF** | **monooxygenase activity** |
| GO:0031406 | MF | carboxylic acid binding | GO:0015318 | MF | inorganic molecular entity transmembrane transporter activity |
| **GO:0004420** | **MF** | **hydroxymethylglutaryl-CoA reductase (NADPH) activity** | **GO:0004420** | **MF** | **hydroxymethylglutaryl-CoA reductase (NADPH) activity** |
| GO:0004222 | MF | metalloendopeptidase activity | GO:0015075 | MF | monoatomic ion transmembrane transporter activity |
| **GO:1990404** | **MF** | **NAD+-protein mono-ADP-ribosyltransferase activity** | GO:0008324 | MF | monoatomic cation transmembrane transporter activity |
| **GO:0003950** | **MF** | **NAD+ poly-ADP-ribosyltransferase activity** | **GO:0046906** | **MF** | **tetrapyrrole binding** |
| GO:0016491 | MF | oxidoreductase activity | **GO:0020037** | **MF** | **heme binding** |
| GO:0004777 | MF | succinate-semialdehyde dehydrogenase (NAD+) activity | GO:0004198 | MF | calcium-dependent cysteine-type endopeptidase activity |
| GO:0009013 | MF | succinate-semialdehyde dehydrogenase [NAD(P)+] activity |  |  |  |
| **GO:0005506** | **MF** | **iron ion binding** |  |  |  |
| **GO:0016705** | **MF** | **oxidoreductase activity, acting on paired donors, with incorporation or reduction of molecular oxygen** |  |  |  |
| GO:0008061 | MF | chitin binding |  |  |  |
| GO:0051117 | MF | ATPase binding |  |  |  |
| **GO:0004497** | **MF** | **monooxygenase activity** |  |  |  |
| **GO:0046906** | **MF** | **tetrapyrrole binding** |  |  |  |
| **GO:0020037** | **MF** | **heme binding** |  |  |  |
| GO:0043531 | MF | ADP binding |  |  |  |
| GO:0008395 | MF | steroid hydroxylase activity |  |  |  |
| GO:0140110 | MF | transcription regulator activity |  |  |  |

^1^GO terms in bold are enriched in both isolates.

^2^Abbreviations: BP: biological process, MF: molecular function.

Supplementary Table S4. Long-read and short-read assemblies used in the analysis of *SIX* genes and race-specific genes.

| **Lineage** | **Accession Number** | **forma specialis and race** | ***SIX8*** | ***SIX9.1*** | ***SIX9.2*** | ***SIX9.3*** | ***SIX9.4*** | ***SIX14*** | **Sequencing technology** | **Assembly accession** |
| --- | --- | --- | --- | --- | --- | --- | --- | --- | --- | --- |
| 2B | 160527 | *Fusarium oxysporum* f. sp. *cubense* |  |  |  |  | X |  | PacBio Sequal | GCA_005930515.1 |
| 2E | Fo5176 | *F. oxysporum* f. sp. *conglutinans* |  |  | X |  | X |  | PacBio Sequal+Novaseq | GCA_014154955.1 |
| 2E | race1 | *F. oxysporum* f. sp. *conglutinans* | X |  | X |  | X |  | PacBio Sequal | GCA_014839635.1 |
| 2E | 39 | *F. oxysporum* f. sp. *lini* |  |  |  |  | X |  | PacBio RS+Illumina | GCA_013423245.1 |
| 2E | Fom005 | *F. oxysporum* f. sp. *melonis* |  |  |  |  |  |  | PacBio | GCA_001703205.2 |
| 2E | 110407-3-1-1 | *F. oxysporum* f. sp. *niveum* |  |  |  |  | X |  | Nanopore MinION | GCA_019593455.1 |
| 2E | Tf1262 | *F. oxysporum* f. sp. *raphani* |  |  | X | X | X |  | PacBio RSII | GCA_019157275.1 |
| 2E | LA3B | *F. oxysporum* f. sp. *vasinfectum* |  |  |  |  |  |  | PacBio RSII | GCA_009602545.1 |
| 2E | TF1 | *F. oxysporum* f. sp. *vasinfectum* race1 |  |  |  |  | X |  | PacBio RSII | GCA_009602505.1 |
| 2G | NRRL38295 | *F. oxysporum* f. sp. *apii* race 3 |  | X | X | X |  |  | PacBio RSII | GCA_014843565.1 |
| 2G | 274AC | *F. oxysporum* f. sp. *apii* race 4 |  | X | X | X |  |  | PacBio RSII | GCA_014843555.1 |
| 2G | Foci3-2 | *F. oxysporum* f. sp. *coriandrii* |  | X | X | X |  |  | PacBio RSII | GCA_014843415.1 |
| 2G | FociGL306 | *F. oxysporum* f. sp. *coriandrii* |  | X | X | X |  |  | PacBio RSII | GCA_014843445.1 |
| 2G | GL1381 | *F. oxysporum* f. sp. *fragariae* |  |  |  |  |  |  | PacBio RSII | GCA_016170095.2 |
| 2G | GL1315 | *F. oxysporum* f. sp. *fragariae* |  |  |  |  |  |  | PacBio RSII+Novaseq | GCA_016166095.1 |
| 2G | BRIP62122a | *F. oxysporum* f. sp. *fragariae* |  |  |  |  |  |  | PacBio Sequal | GCA_016166325.2 |
| 2G | MAFF7271510 | *F. oxysporum* f. sp. *fragariae* |  |  |  |  |  |  | PacBio Sequal | GCA_016164145.2 |
| 2G | GL1080 | *F. oxysporum* f. sp. *fragariae* |  |  |  |  |  |  | PacBio RSII | GCA_016170085.2 |
| 2G | 150523 | *F. oxysporum* f. sp. *niveum* | X | X | X | X | X | X | Nanopore +MinION | GCA_019593445.1 |
| 2G | 150524 | *F. oxysporum* f. sp. *niveum* | X | X | X | X | X | X | Nanopore MinION | GCA_019593505.1 |
| 2G | MR4003 | *F. oxysporum* f. sp. *sesami* | X |  | X | X | X |  | PacBio Sequal | GCA_017979615.1 |
| 2G | LA1E | *F. oxysporum* f. sp. *vasinfectum* |  |  |  |  |  |  | PacBio RSII | GCA_009602615.1 |
| 2G | 89-1A | *F. oxysporum* f. sp. *vasinfectum* race 4 |  | X |  | X | X |  | PacBio RSII | GCA_009602635.1 |
| 3B | 14-004 | *F. oxysporum* f. sp. *vasinfectum* |  |  |  |  |  |  | PacBio RSII | GCA_009602625.1 |
| 3D | Fol4287 | *F. oxysporum* f. sp. *lycopersici* race 2 | X | X |  |  |  | X | Pacbio Hifi+NovaSeq | GCA_000149955.2 |
| 3D | D11 | *F. oxysporum* f. sp. *lycopersici* race 3 | X | X |  |  |  | X | PacBio RSII | GCA_003977725.1 |
| 3D | 26406 | *F. oxysporum* f. sp. *melonis* |  |  |  |  |  |  | PacBio | GCA_002318975.1 |
| 3F | 207-A | *F. oxysporum* f. sp. *apii* race 2 |  | X |  |  |  |  | PacBio RSII | GCA_014843455.1 |
| 3F | 2a | *F. oxysporum* f. sp. *lactucae* race 1 |  |  |  |  | X | X | Hiseq | in-house |
| 3F | 20a | *F. oxysporum* f. sp. *lactucae* race 1 |  |  |  |  | X | X | Hiseq | in-house |
| 3F | 45a | *F. oxysporum* f. sp. *lactucae* race 1 |  |  |  |  | X | X | Hiseq | in-house |
| 3F | 50a | *F. oxysporum* f. sp. *lactucae* race 1 |  |  |  |  | X | X | Hiseq | in-house |
| 3F | 52a | *F. oxysporum* f. sp. *lactucae* race 1 |  |  |  |  | X | X | Hiseq | in-house |
| 3F | 53a | *F. oxysporum* f. sp. *lactucae* race 1 |  |  |  |  | X | X | Hiseq | in-house |
| 3F | 58a* | *F. oxysporum* f. sp. *lactucae* race 1 |  |  |  |  | X | X | Hiseq | in-house |
| 3F | 74a | *F. oxysporum* f. sp. *lactucae* race 1 |  |  |  |  | X | X | Hiseq | in-house |
| 3F | 6-14 | *F. oxysporum* f. sp. *lactucae* race 1 |  |  |  |  | X | X | Hiseq | in-house |
| 3F | 7-14 | *F. oxysporum* f. sp. *lactucae* race 1 |  |  |  |  | X | X | Hiseq | in-house |
| 3F | AC013 | *F. oxysporum* f. sp. *lactucae* race 1 |  |  |  |  | X | X | Hiseq | in-house |
| 3F | AJ520* | *F. oxysporum* f. sp. *lactucae* race 1 |  |  |  |  | X | XX | Nanopore MinION+Miseq | GCA_045838095.1 |
| 3F | AJ718* | *F. oxysporum* f. sp. *lactucae* race 1 |  |  |  |  | X | XX | Nanopore MinION | GCA_045837815.1 |
| 3F | AJ865* | *F. oxysporum* f. sp. *lactucae* race 1 |  |  |  |  | X | XX | Nanopore MinION | GCA_045837925.1 |
| 3F | AL010 | *F. oxysporum* f. sp. *lactucae* race 1 |  |  |  |  | X | X | Hiseq | in-house |
| 3F | AM163 | *F. oxysporum* f. sp. *lactucae* race 1 |  |  |  |  | X | X | Hiseq | in-house |
| 3F | AP057 | *F. oxysporum* f. sp. *lactucae* race 1 |  |  |  |  | X | X | Hiseq | in-house |
| 3F | AP068 | *F. oxysporum* f. sp. *lactucae* race 1 |  |  |  |  | X | X | Hiseq | in-house |
| 3F | AS147 | *F. oxysporum* f. sp. *lactucae* race 1 |  |  |  |  | X | X | Hiseq | in-house |
| 3F | AT142* | *F. oxysporum* f. sp. *lactucae* race 1 |  |  |  |  | X | X | **Nanopore PromethION+**Hiseq | in-house |
|  |  |  |  |  |  |  |  |  |  |  |
| 3F | GL1546 | *F. oxysporum* f. sp. *lactucae* race 1 |  |  |  |  | X | X | Hiseq | in-house |
| 3F | GL1692 | *F. oxysporum* f. sp. *lactucae* race 1 |  |  |  |  | X | X | Hiseq | in-house |
| 3F | GL1693 | *F. oxysporum* f. sp. *lactucae* race 1 |  |  |  |  | X | X | Hiseq | in-house |
| 3F | GL1815 | *F. oxysporum* f. sp. *lactucae* race 1 |  |  |  |  | X | X | Hiseq | in-house |
| 3F | JCP024 | *F. oxysporum* f. sp. *lactucae* race 1 |  |  |  |  | X | X | Hiseq | in-house |
| **3F** | **JCP043*** | ***F. oxysporum* f. sp. *lactucae* race 1** |  |  |  |  | **X** | **X** | **Nanopore PromethION+PacBio HiFi+Hiseq** | **in-house** |
| 3F | JCP053 | *F. oxysporum* f. sp. *lactucae* race 1 |  |  |  |  | X | X | Hiseq | in-house |
| 3F | JCP293 | *F. oxysporum* f. sp. *lactucae* race 1 |  |  |  |  | X | X | Hiseq | in-house |
| 3F | JCP360 | *F. oxysporum* f. sp. *lactucae* race 1 |  |  |  |  | X | X | Hiseq | in-house |
| 3F | JCP381 | *F. oxysporum* f. sp. *lactucae* race 1 |  |  |  |  | X | X | Hiseq | in-house |
| 3F | Mya | *F. oxysporum* f. sp. *lactucae* race 1 |  |  |  |  | X | X | Hiseq | in-house |
| 3F | SB1-1 | *F. oxysporum* f. sp. *lactucae* race 1 |  |  |  |  | X | X | Hiseq | in-house |
| 3F | Fol321 | *F. oxysporum* f. sp. *lactucae* race 1 |  |  |  |  | X | X | Novaseq | GCA_045786965.1 |
| 3F | VSP-0794 | *F. oxysporum* f. sp. *lactucae* race 1 |  |  |  |  | X | X | Novaseq | GCA_045786735.1 |
| 3F | Fol621s | *F. oxysporum* f. sp. *lactucae* race 1 variant |  |  |  |  | X | X | Novaseq | GCA_045786925.1 |
| 3F | VSP-0916 | *F. oxysporum* f. sp. *lactucae* race 1 variant |  |  |  |  | X | X | Novaseq | GCA_045786885.1 |
| 1 | F9501* | *F. oxysporum* f. sp. *lactucae* race 2 | X | X | X | X | X | X | Hiseq | in-house |
| 2G | FLK1001* | *F. oxysporum* f. sp. *lactucae* race 3 | X | X | X | X | X | X | Hiseq | in-house |
| 3F | 888 | *F. oxysporum* f. sp. *lactucae* race 4 | X | X | X |  | X | X | Hiseq | in-house |
| 3F | AJ516* | *F. oxysporum* f. sp. *lactucae* race 4 | X |  | X | X | XX | X | Nanopore MinION+Miseq | GCA_045838095.1 |
| 3F | AJ592* | *F. oxysporum* f. sp. *lactucae* race 4 | X | X | X | X | XX | X | Nanopore MinION | GCA_045838035.1 |
| 3F | AJ705* | *F. oxysporum* f. sp. *lactucae* race 4 | X | X | X | X | XX | X | Nanopore MinION | GCA_045837975.1 |
| 3F | AL088 | *F. oxysporum* f. sp. *lactucae* race 4 | X | X |  | X | X | X | Hiseq | in-house |
| 3F | AL127 | *F. oxysporum* f. sp. *lactucae* race 4 | X |  | X |  | X | X | Hiseq | in-house |
| 3F | AL185 | *F. oxysporum* f. sp. *lactucae* race 4 | X |  | X | X | X | X | Hiseq | in-house |
| 3F | AM020 | *F. oxysporum* f. sp. *lactucae* race 4 | X |  | X | X | X | X | Hiseq | in-house |
| 3F | AN072 | *F. oxysporum* f. sp. *lactucae* race 4 | X |  | X |  | X | X | Hiseq | in-house |
| 3F | AP004 | *F. oxysporum* f. sp. *lactucae* race 4 | X |  | X | X | X | X | Hiseq | in-house |
| 3F | AP114 | *F. oxysporum* f. sp. *lactucae* race 4 | X | X | X | X | X | X | Hiseq | in-house |
| **3F** | **AT141*** | ***F. oxysporum* f. sp. *lactucae* race 4** | **XX** |  | **X** | **X** | **XX** | **X** | **Nanopore PromethION+PacBio HiFi+Hiseq** | **in-house** |
| 3F | AU042 | *F. oxysporum* f. sp. *lactucae* race 4 | X |  | X | X | X | X | Novaseq | in-house |
| 3F | AU069 | *F. oxysporum* f. sp. *lactucae* race 4 | X |  | X | X | X | X | Hiseq | in-house |
| 3F | AU078 | *F. oxysporum* f. sp. *lactucae* race 4 | X |  | X | X | X | X | Hiseq | in-house |
| 3F | AU122 | *F. oxysporum* f. sp. *lactucae* race 4 | X |  | X | X | X | X | Hiseq | in-house |
| 3F | R4 | *F. oxysporum* f. sp. *lactucae* race 4 | X | X | X | X | X | X | Hiseq | in-house |
| 3G | FoC_Fus2 | *F. oxysporum* f. sp. *cepae* |  |  |  |  | X |  | PacBio+Miseq | GCA_003615085.1 |
| 3G | Fol001 | *F. oxysporum* f. sp. *lycopersici* race 1 | X | X |  |  |  | X | PacBio RSII | GCA_015345885.1 |
| 3G | Fol010 | *F. oxysporum* f. sp. *lycopersici* race 1 | X | X |  |  |  | X | PacBio RSII | GCA_015345895.1 |
| 3G | Forc016 | *F. oxysporum* f. sp. *radicis cucumerinum* |  |  |  |  | X |  | PacBio | GCA_001702695.2 |
| 3G | Fo47 | tomato endophyte |  |  |  |  |  |  | PacBio RSII | GCA_013085055.1 |

Note: The T2T assemblies of JCP043 and AT141 are marked in bold. FOLac isolates used in the analysis of race-specific genes were indicated with asterisks. X and blank indicate the presence and absence of individual *SIX* genes in each isolate, respectively. XX indicate two copies.

Supplementary Table S5. Detailed information of several specific gene classes that are race 1 and race 4 specific based on the analysis of nine FOLac genomes.

| **Race 1 specific** | | | | | | |
| --- | --- | --- | --- | --- | --- | --- |
| **Category** | **Gene ID** | **Chr** | **Start** | **End** | **Strand** | **Functional annotation** |
| CAZYme | JCP043_012455 | chr7 | 1453573 | 1,456,232 | - | GH3 |
| CAZYme and Effector | JCP043_017764 | chr12 | 626789 | 629,745 | + | CE5 and Apoplastic effector |
| CAZYme | JCP043_017955 | chr12 | 1451264 | 1,453,860 | - | AA3 |
| CAZYme | JCP043_021024 | chr16 | 1219710 | 1221497 | - | CBM21 |
| Effector | JCP043_010340 | chr5 | 4134367 | 4135346 | + | Apoplastic/cytoplasmic effector |
| Effector | JCP043_012389 | chr7 | 1144333 | 1144704 | - | Cytoplasmic effector |
| Effector | JCP043_012449 | chr7 | 1443332 | 1443700 | + | Apoplastic effector |
| Effector | JCP043_012626 | chr7 | 2246400 | 2246934 | - | Apoplastic effector |
| Effector | JCP043_012900 | chr7 | 3789501 | 3790539 | + | Cytoplasmic effector |
| Effector | JCP043_016282 | chr10 | 1812247 | 1813744 | + | Apoplastic effector |
| Effector | JCP043_016411 | chr10 | 2853039 | 2853428 | - | Apoplastic effector |
| Effector | JCP043_017967 | chr12 | 1510074 | 1512094 | + | Apoplastic effector |
| Effector | JCP043_018264 | chr12 | 2633223 | 2633608 | + | Apoplastic effector |
| Effector | JCP043_018319 | chr13 | 74964 | 75482 | - | Cytoplasmic effector |
| Effector | JCP043_018324 | chr13 | 85444 | 86717 | + | Apoplastic/cytoplasmic effector |
| Effector | JCP043_019457 | chr14 | 380320 | 381264 | + | Cytoplasmic effector |
| Effector | JCP043_020515 | chr15 | 913785 | 916174 | - | Apoplastic/cytoplasmic effector |
| Mimp effector | JCP043_010354 | chr5 | 4230880 | 4232159 | - |  |
| Mimp effector | chr5-rna:2834 | chr5 | 4231773 | 4232159 | - |  |
| Mimp effector | JCP043_012900 | chr7 | 3789501 | 3790539 | + |  |
| Mimp effector | chr15-rna:1564 | chr15 | 772953 | 773084 | - |  |
| Mimp effector | chr15-rna:1814 | chr15 | 1627088 | 1627255 | - |  |
| SM | JCP043_010116 | chr5 | 3068339 | 3069849 | - | Hypothetical protein, putatively involved in indole biosynthesis |
| SM | JCP043_017585 | chr11 | 2803794 | 2806167 | - | Hypothetical protein, putatively involved in terpene biosynthesis |
| SM | JCP043_020473 | chr15 | 455347 | 455798 | + | Hypothetical protein, putatively involved in cytokinin biosynthesis |
| **Race 4 specific** | | | | | | |
| **Category** | **Gene ID** | **Chr** | **Start** | **End** | **Strand** | **Functional annotation** |
| CAZYme | AT141_017965 | Chr13 | 342591 | 343655 | + | CE16 |
| CAZYme and Effector | AT141_017966 | Chr13 | 344580 | 346134 | - | GH28 and Apoplastic effector |
| Effector | AT141_000011 | Chr1 | 72061 | 72558 | - | Apoplastic effector |
| Effector | AT141_006275 | Chr4 | 122737 | 123096 | + | Apoplastic effector |
| Effector | AT141_017919 | Chr13 | 113750 | 113992 | - | Apoplastic/cytoplasmic effector |
| Effector | AT141_018035 | Chr13 | 752272 | 752835 | + | Apoplastic/cytoplasmic effector |
| Effector | AT141_018150 | Chr13 | 1402276 | 1402842 | + | Apoplastic effector |
| Effector | AT141_020054 | Chr16 | 1650617 | 1650878 | + | Apoplastic effector |
| Effector | AT141_020387 | Chr18 | 202461 | 202752 | + | Apoplastic effector |
| Mimp effector | Chr5-rna:2843 | Chr5 | 4223968 | 4224519 | + |  |
| Mimp effector | Chr15-rna:980 | Chr15 | 1714414 | 1714719 | + |  |
| Mimp effector | Chr16-rna:1201 | Chr16 | 1253114 | 1253317 | + |  |
| SM | AT141_012629 | Chr8 | 390623 | 393087 | + | Hypothetical protein, putatively involved in terpene biosynthesis |
| SM | AT141_019910 | Chr16 | 900867 | 903575 | + | GH18 |
| SM | AT141_019912 | Chr16 | 908238 | 908843 | + | Putative type-III polyketide synthase |
| SM | AT141_019913 | Chr16 | 913480 | 915093 | + | Phosphate transporter |
| SM | AT141_019917 | Chr16 | 933842 | 934143 | + | Hypothetical protein |
| SM | AT141_019918 | Chr16 | 934938 | 937413 | - | Hypothetical protein |

Note: Nucleotide sequences of the race-specific genes are provided in Supplementary File S5.


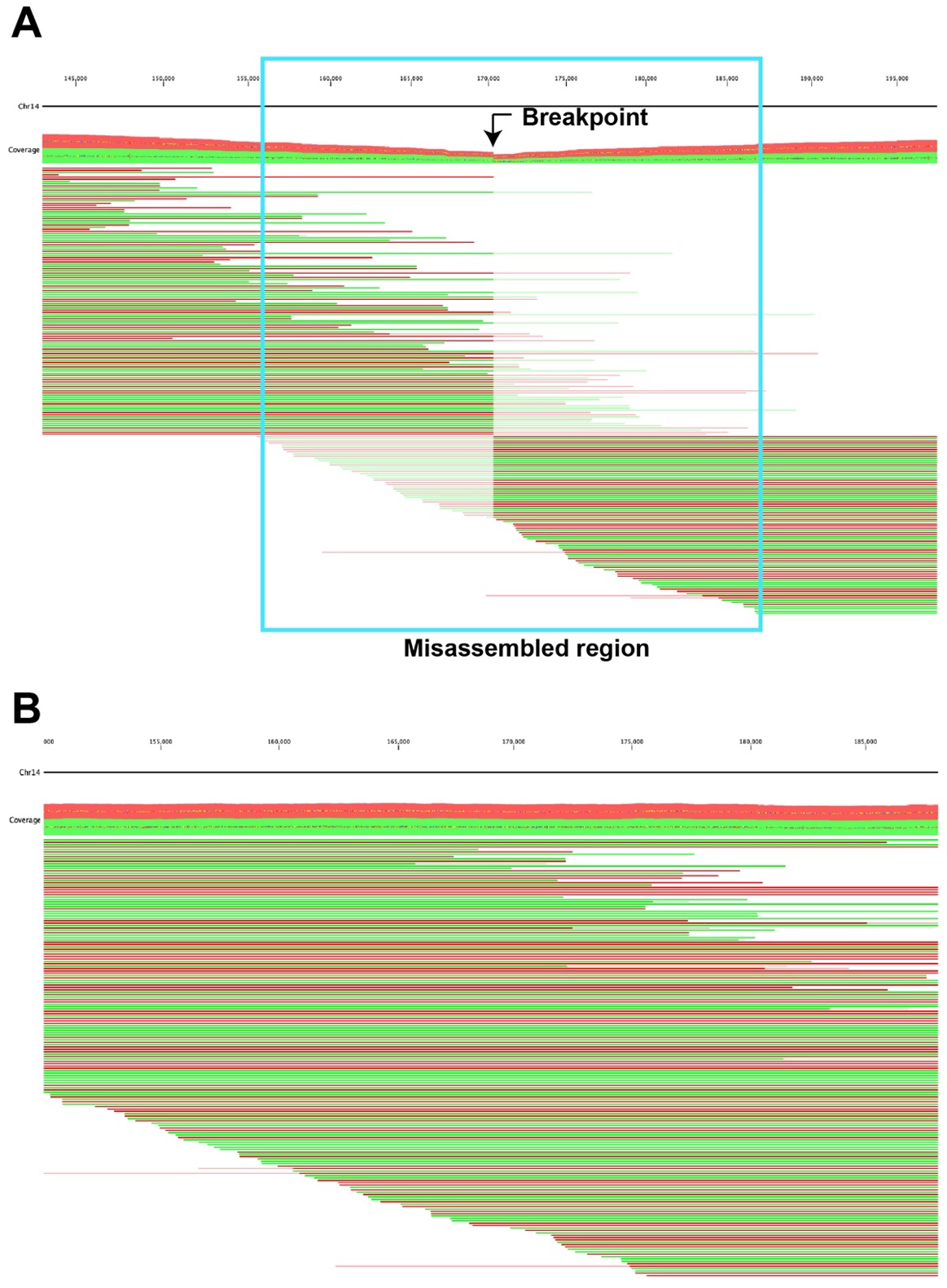


Supplementary Figure S1. ONT reads mapping to the NECAT assembly of AT141 before and after correction was made on the misassembled region. (A) Misassembled region marked in blue box was identified based on a sharp decrease in read depth with a breakpoint marked with an arrow. Mismatches in reads are indicated with light colors. Correction was made by splitting the contig into two pieces at the breakpoint, followed by end extension and rejoining. (B) The updated version of the contig was confirmed to be accurate based on uniform and continuous read coverage. Reads coded in red and green indicate forward and reverse directions, respectively.


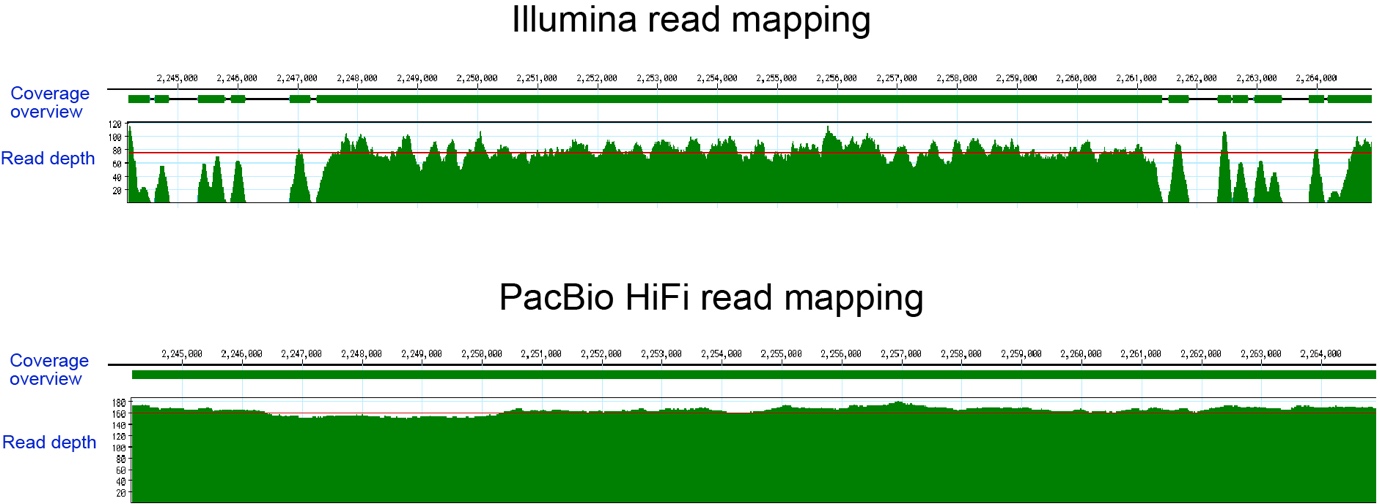


Supplementary Figure S2. Comparison of Illumina (top panel) vs. PacBio HiFi reads (bottom panel) mapping to the same accessory region of JCP043. Illumina reads displayed low and zero read coverage in multiple regions, whereas continuous and uniform read coverage was observed using PacBio HiFi reads.


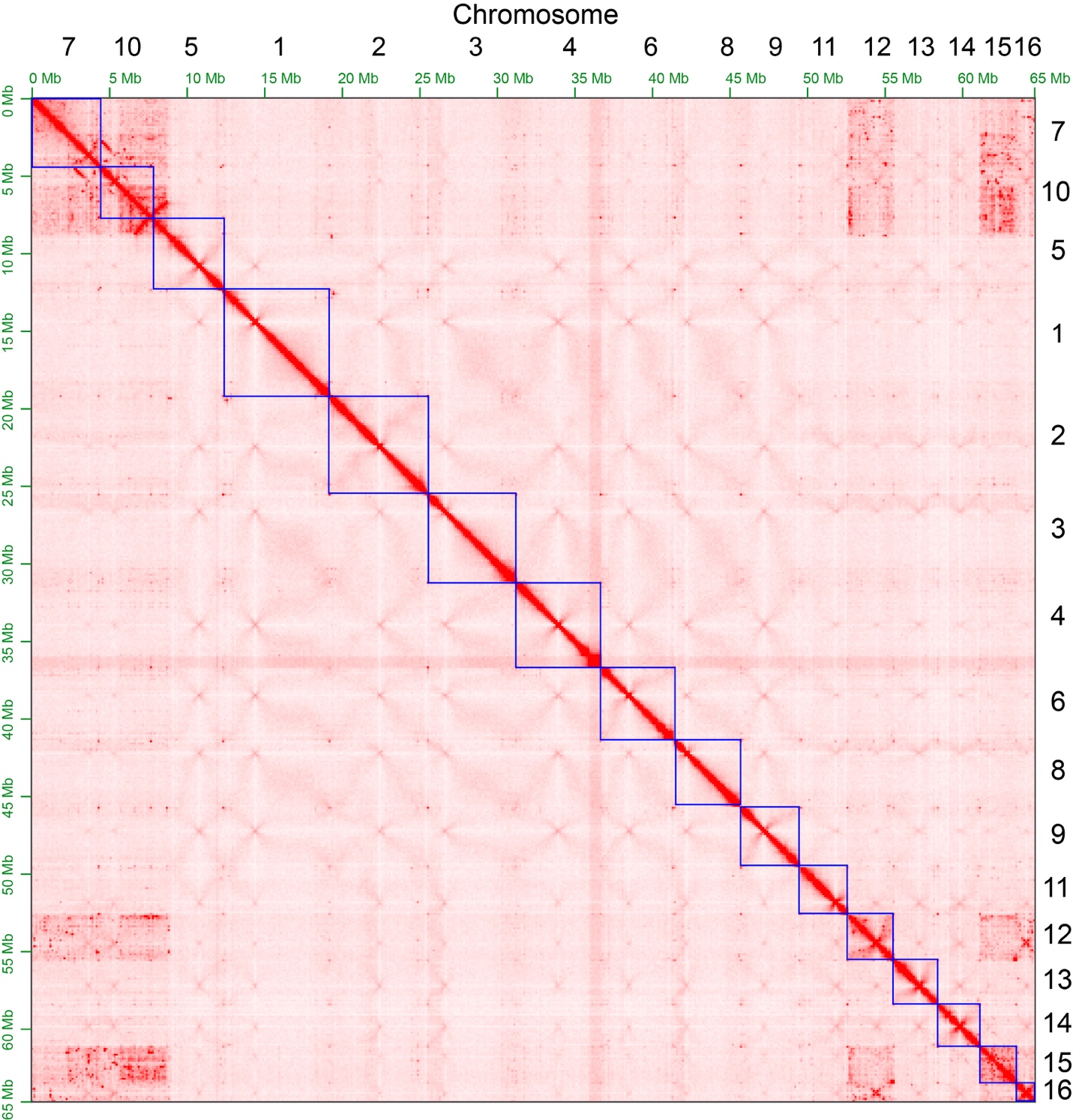


Supplementary Figure S3**.** Genome-wide Hi-C contact map showing the interaction matrices among 16 chromosomes. The red bright diagonal band within each square represents elevated contact frequency along the chromosome, indicating correct chromosome organization.

Supplementary Figure 4A


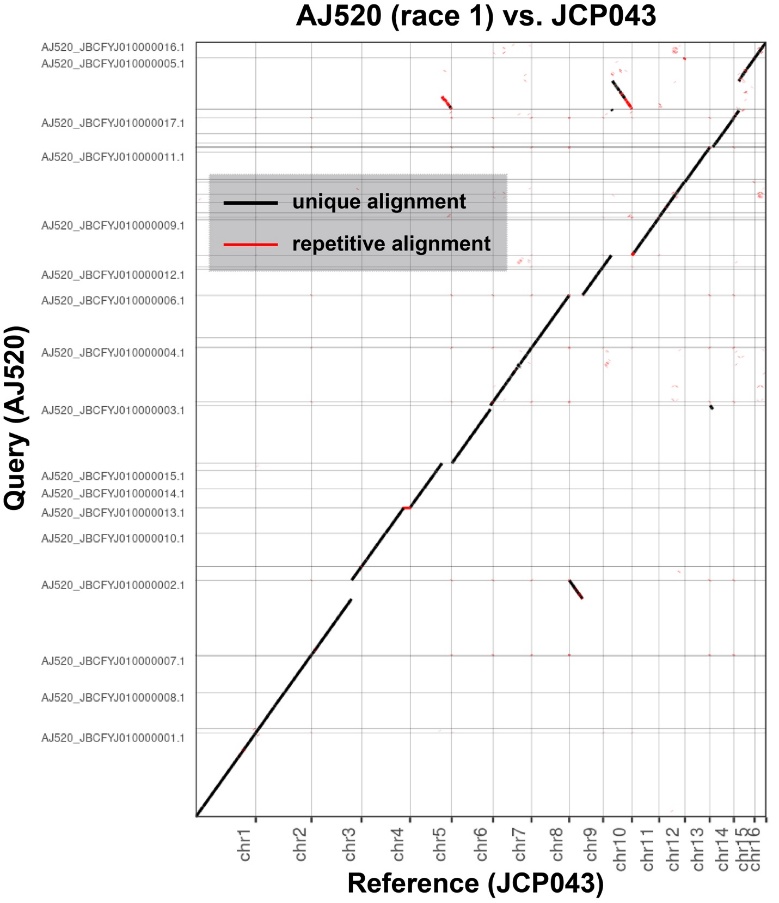


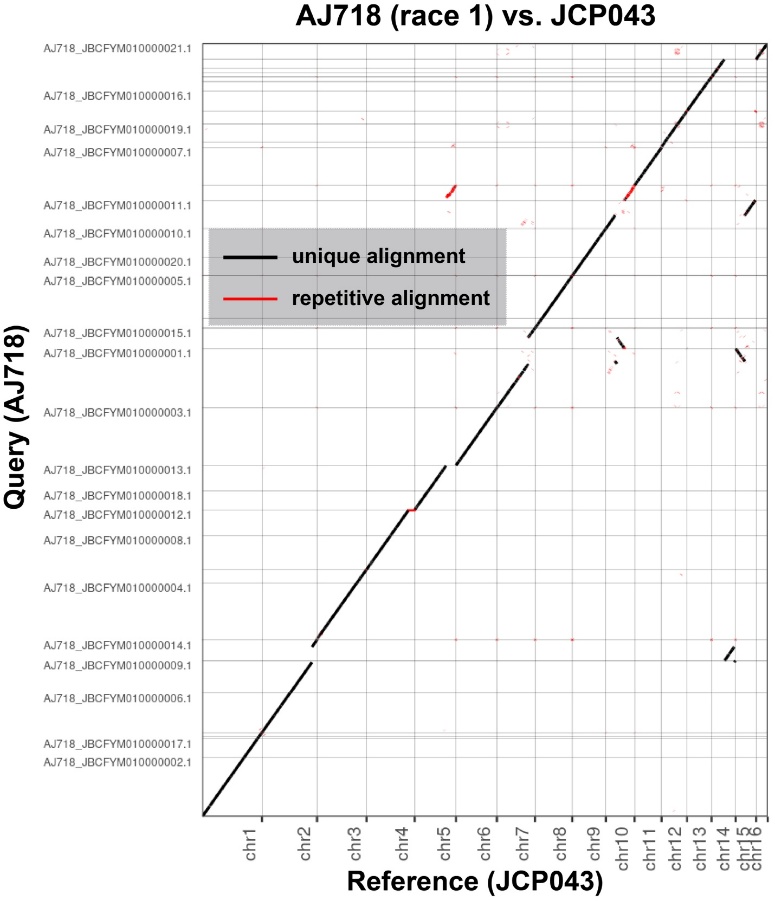


Supplementary Figure 4A


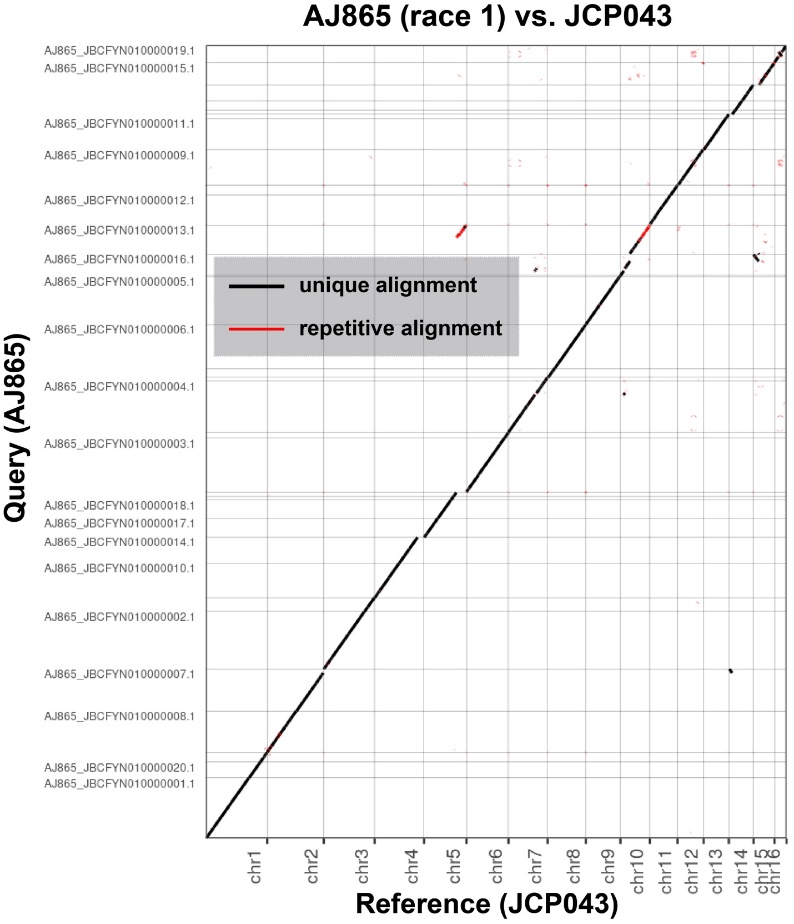


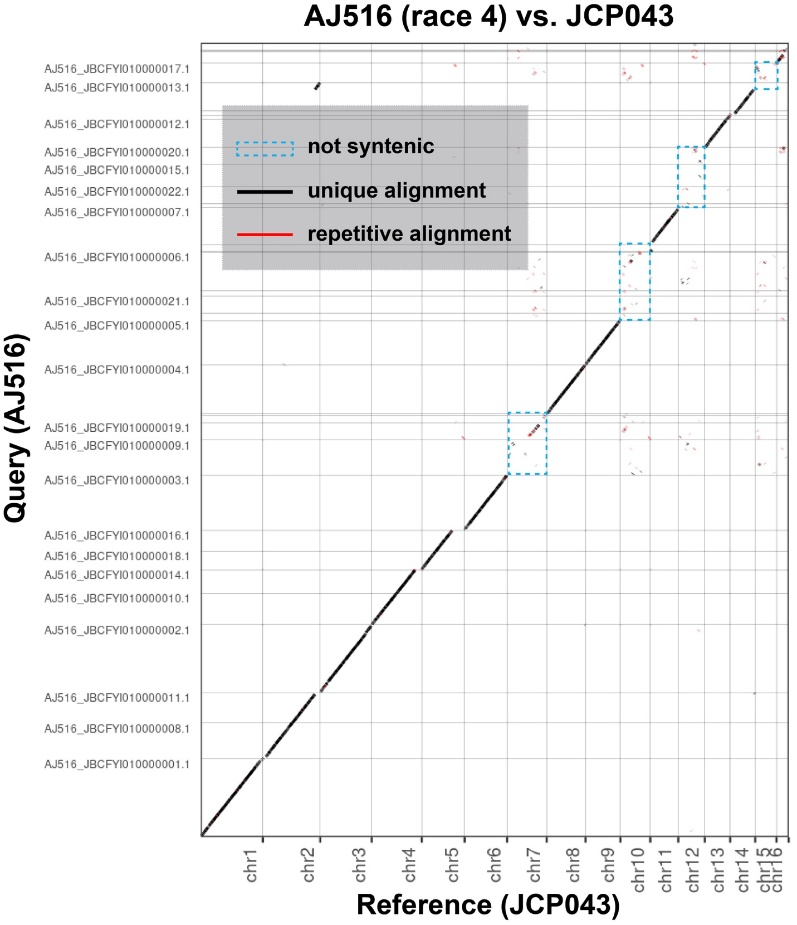


Supplementary Figure 4A


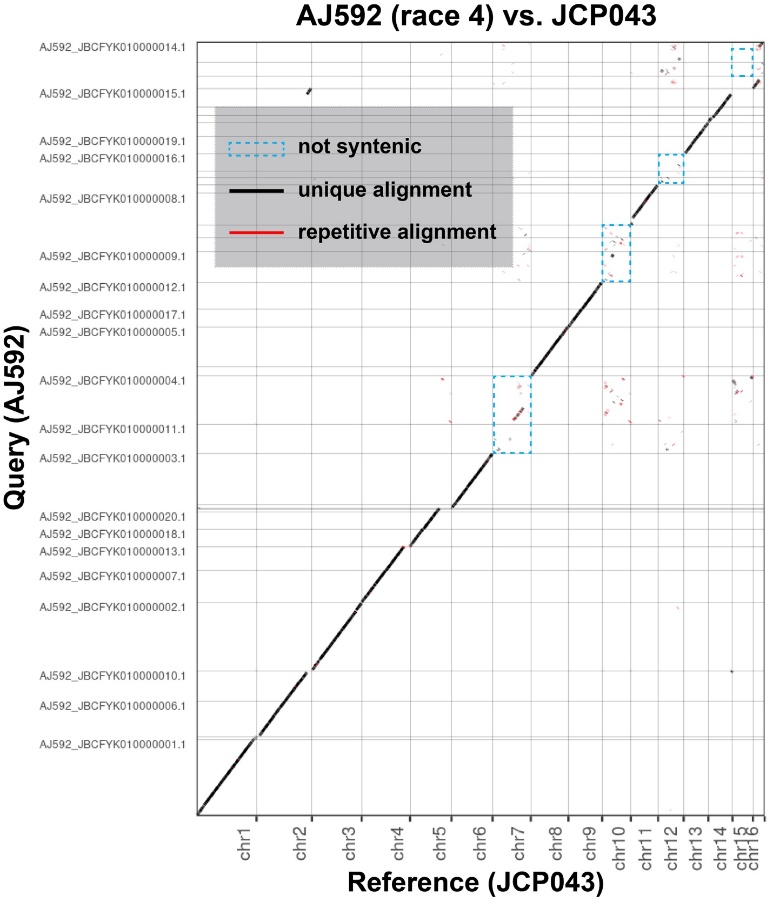

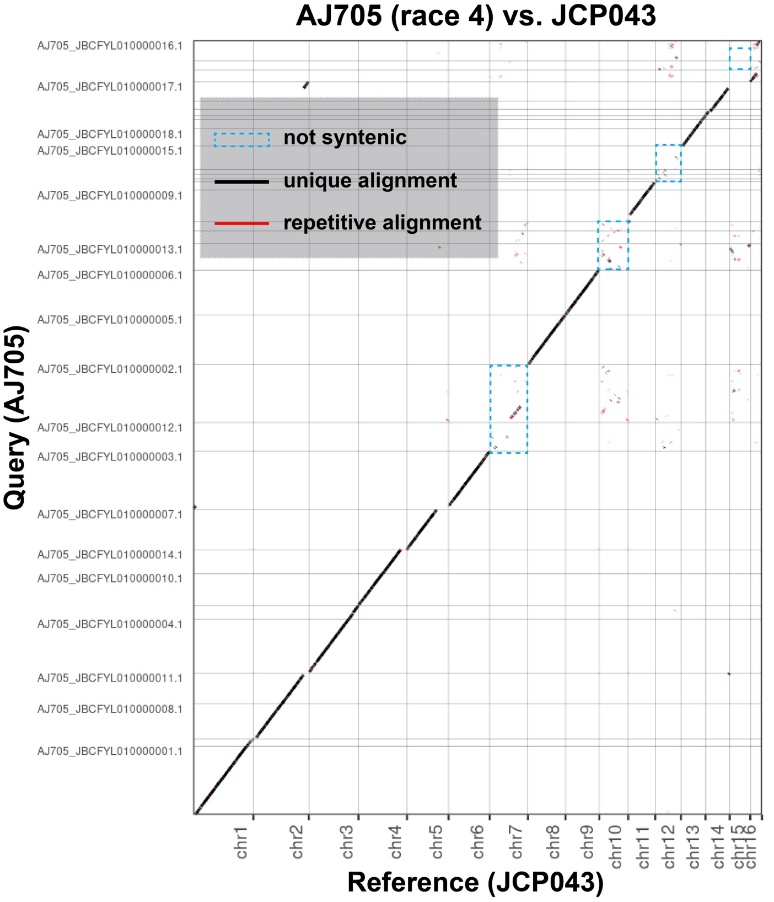


Supplementary Figure 4B


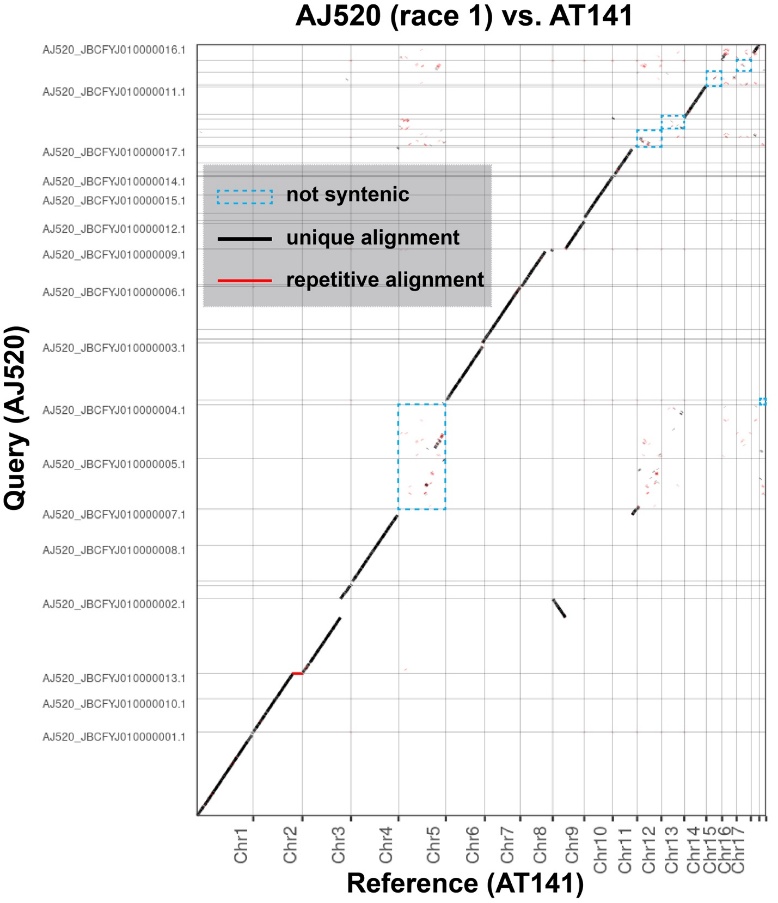


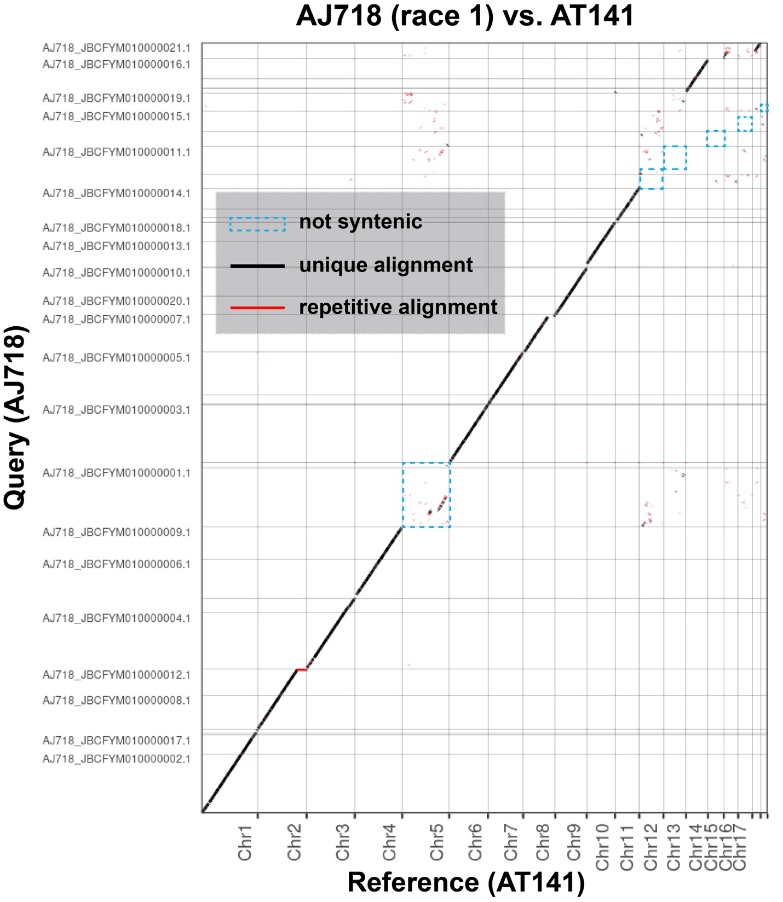


Supplementary Figure 4B


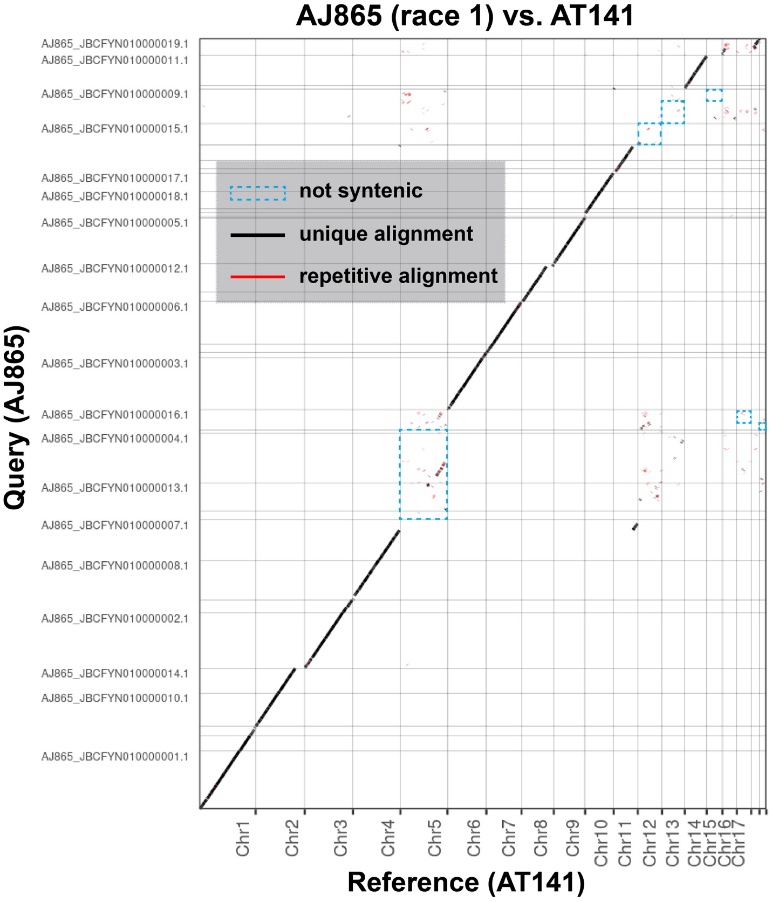


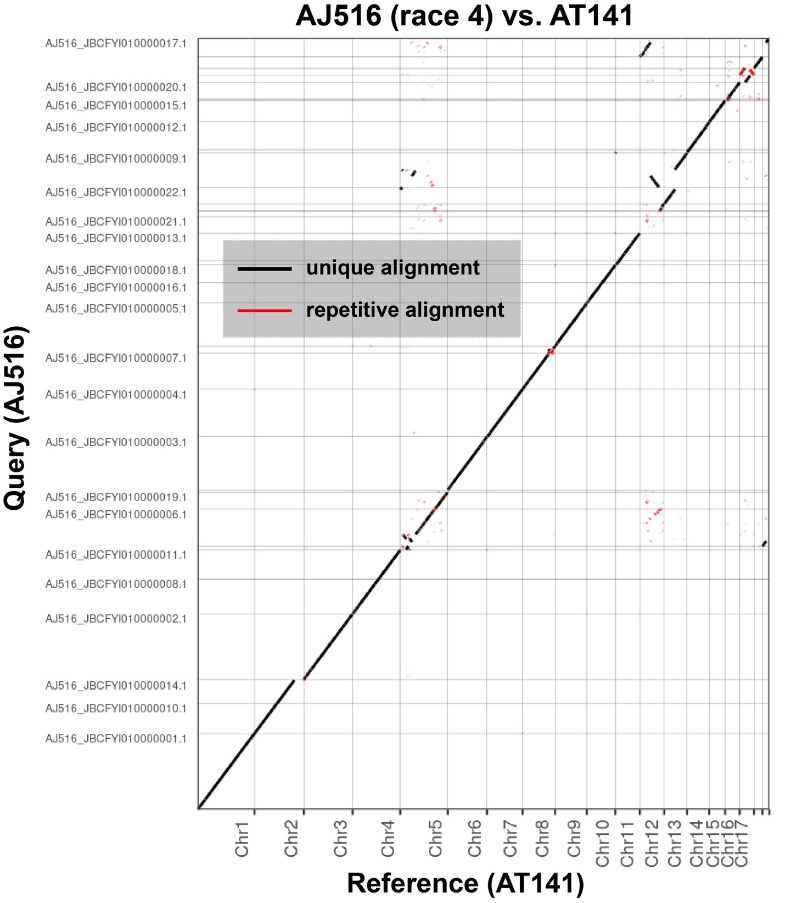


Supplementary Figure 4B


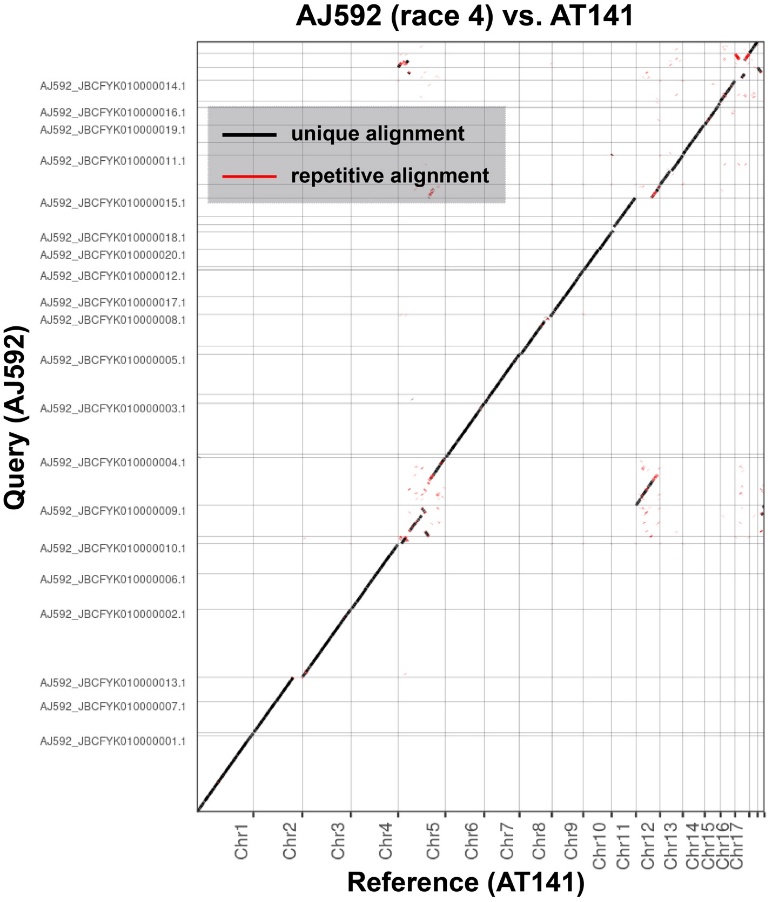


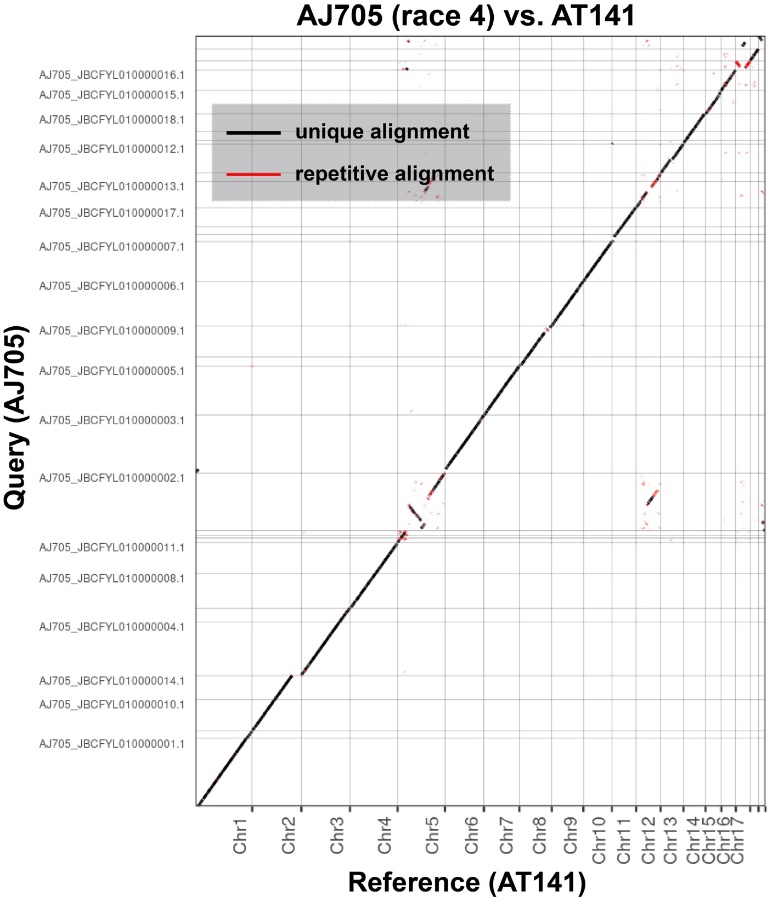


Supplementary Figure S4. Dot plots depicting the synteny between the T2T assemblies for race 1 and 4 isolates reported herein and each of the six published FOLac race 1 and 4 assemblies (Bastes et al. 2024). The nucmer package (nucmer –maxmatch; -L 10000) was used to identify highly similar regions, followed by data visualization with Assemblytics (Nattestad and Schatz 2016). (**A**) Race 1 JCP043 (Reference) accessory chromosomes 7, 10, 12 and 15 showed great synteny with the three FOLac race 1 isolates but not with the three FOLac race 4 isolates, which are highlighted in blue dotted rectangular boxes. (**B**) Race 4 AT141 (Reference) accessory chromosomes 5, 12, 13, 15, 17, and 19 showed great synteny with the three FOLac race 4 isolates but not with the three FOLac race 1 isolates, which are highlighted in blue dotted rectangular boxes.


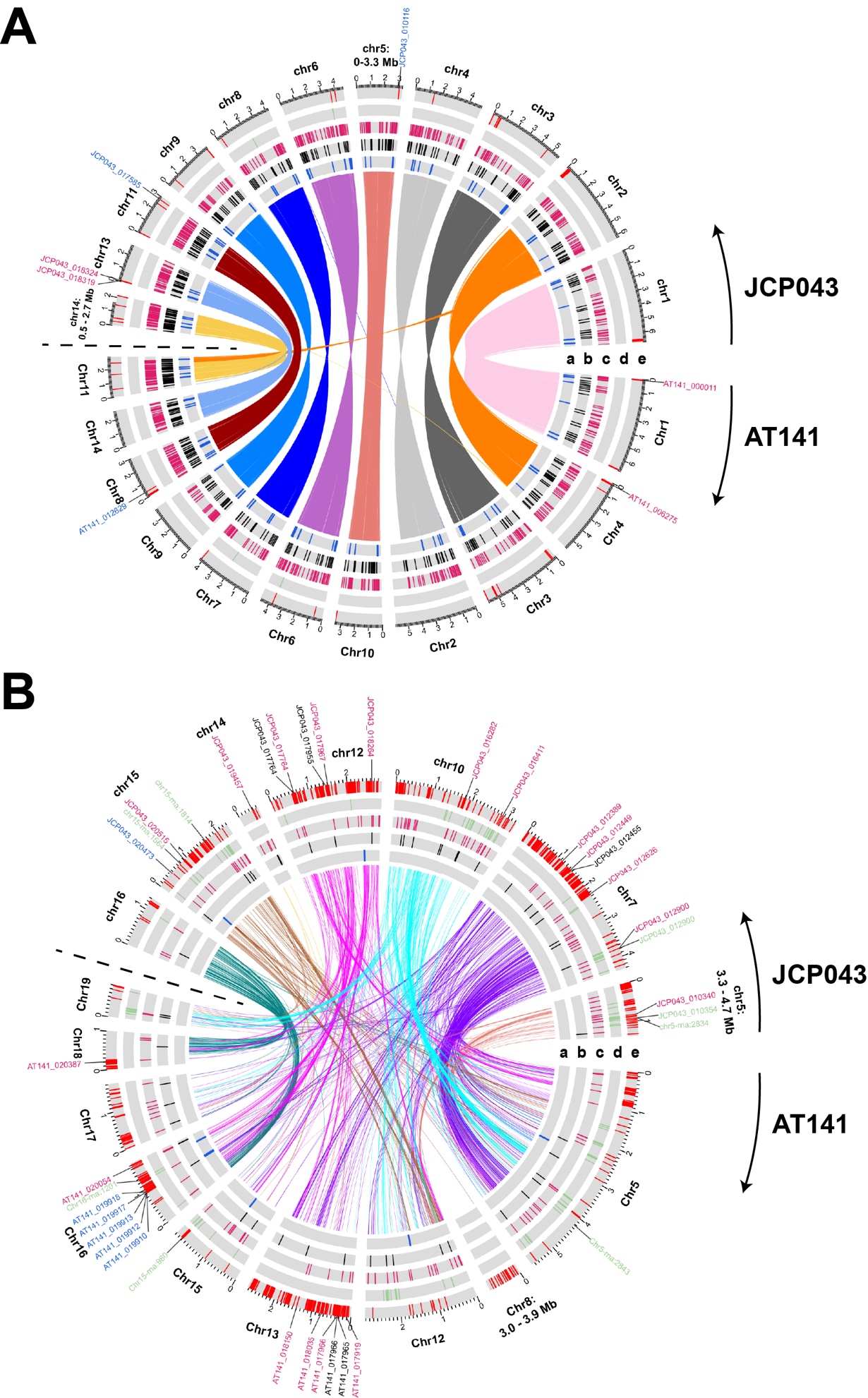


Supplementary Figure S5. Visualization of homologous gene pairs and the distribution of virulence and race-specific genes in the (A) core and (B) accessory genomes of JCP043 and AT141. Order of tracks from inward to outward (a-e) represent the location of: a: secondary metabolite (SM) gene clusters, b: secreted carbohydrate-activate enzymes (CAZYmes), c: effector genes, d: *mimp*-associated effector genes, and e: race-specific genes. Gene IDs of the race-specific virulence genes, including SM genes (blue), secreted CAZYmes (black), effectors (pink), and *mimp*-associated effectors (green), are shown outside of track e. See Supplementary Table S3 for detailed information of the race-specific virulence genes. Lines in the center represent pairs of homologous genes (15063 pairs in the core; 917 pairs in the accessory) identified with reciprocal best BLAST hit.

Variant calling criteria: Variant is present in >=70% of the mapped reads (with 99% sequence identity and read depth of >=3)


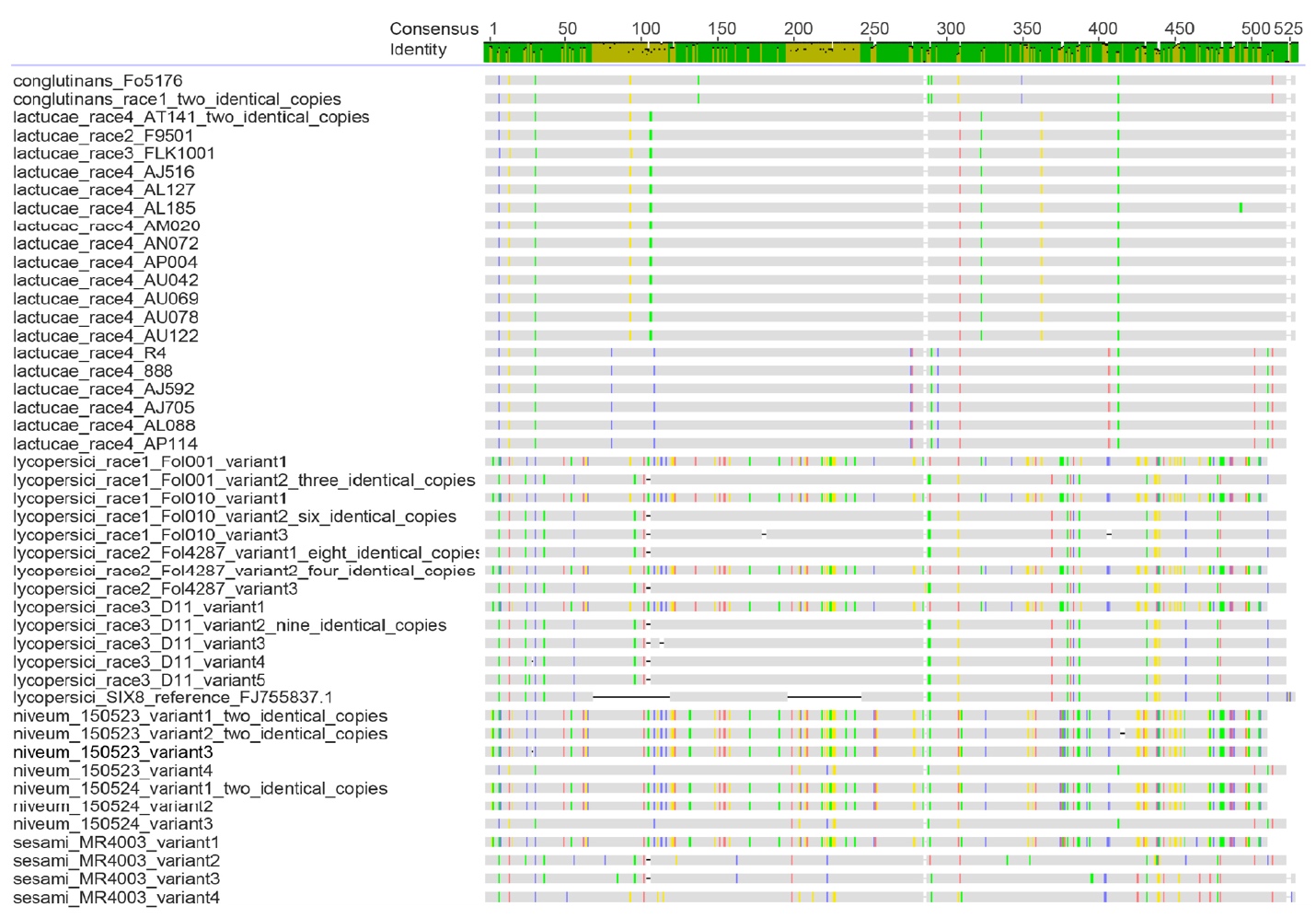


Supplementary Figure S6. MUSCLE alignment of *SIX8* sequences from *F. oxysporum ff. spp. conglutinans, lactucae, lycopersici,* and *niveum.* Nucleotide with 100% identity among all the sequences are indicated with grey bars, whereas gaps and indels are indicated with horizontal lines. Single nucleotide polymorphisms are noted with colored lines. Identity scale for the consensus sequence shown on the top: green=100%, gold=30-99.9%.

**
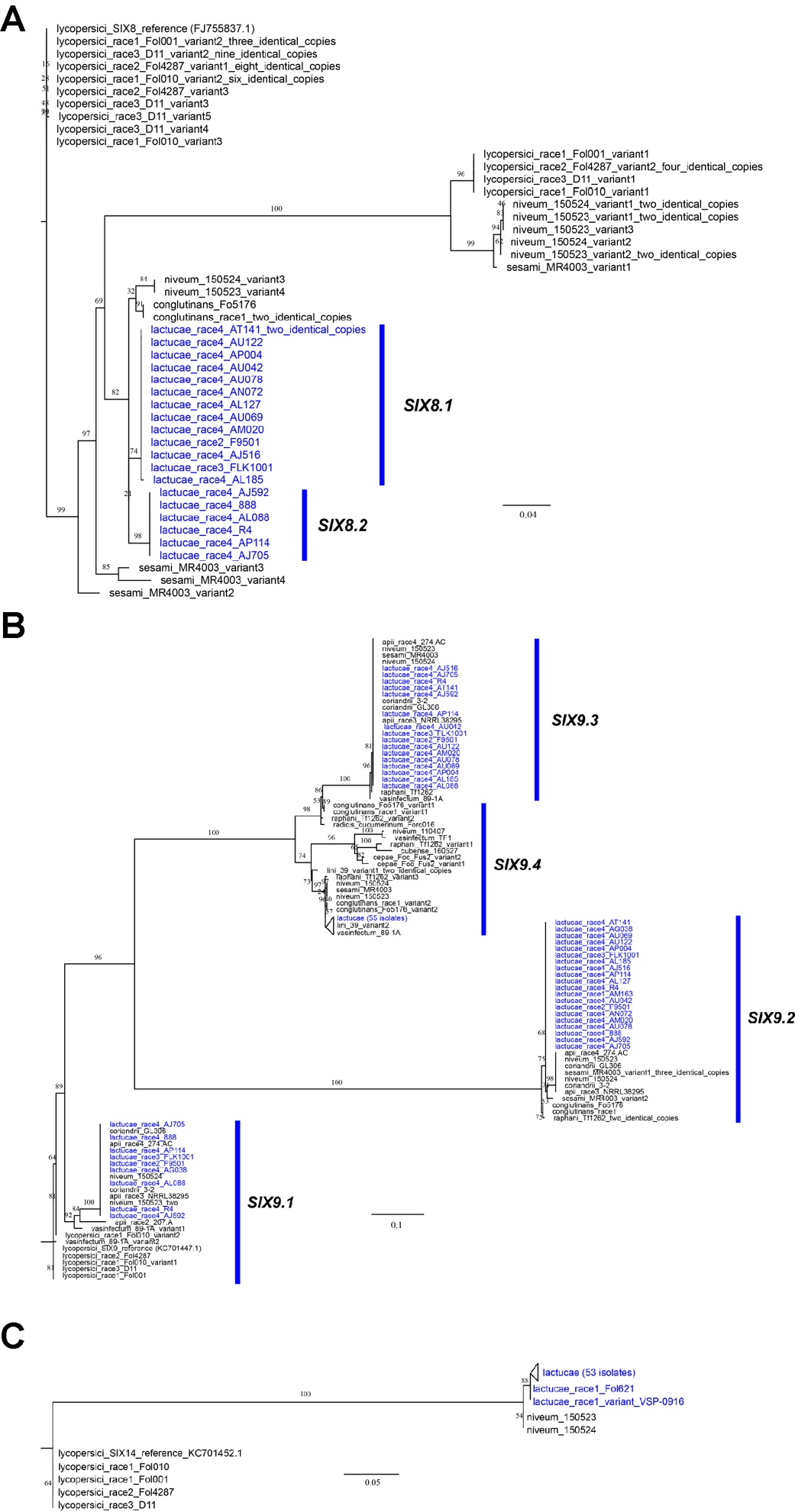
**

Supplementary Figure S7. Phylogenetic analysis of *SIX8, SIX9,* and *SIX14* using 87 *F. oxysporum* genomes. Maximum likelihood trees of (**A**) *SIX8*, (**B**) *SIX9*, and (**C**) *SIX14*, inferred using IQ-TREE 2 [89]. Each tree is rooted through the *F. oxysporum* f. sp. *lycopersici SIX* gene references. The numbers above the branches indicate bootstrap values. Taxon names of FOLac isolates are highlighted in blue.


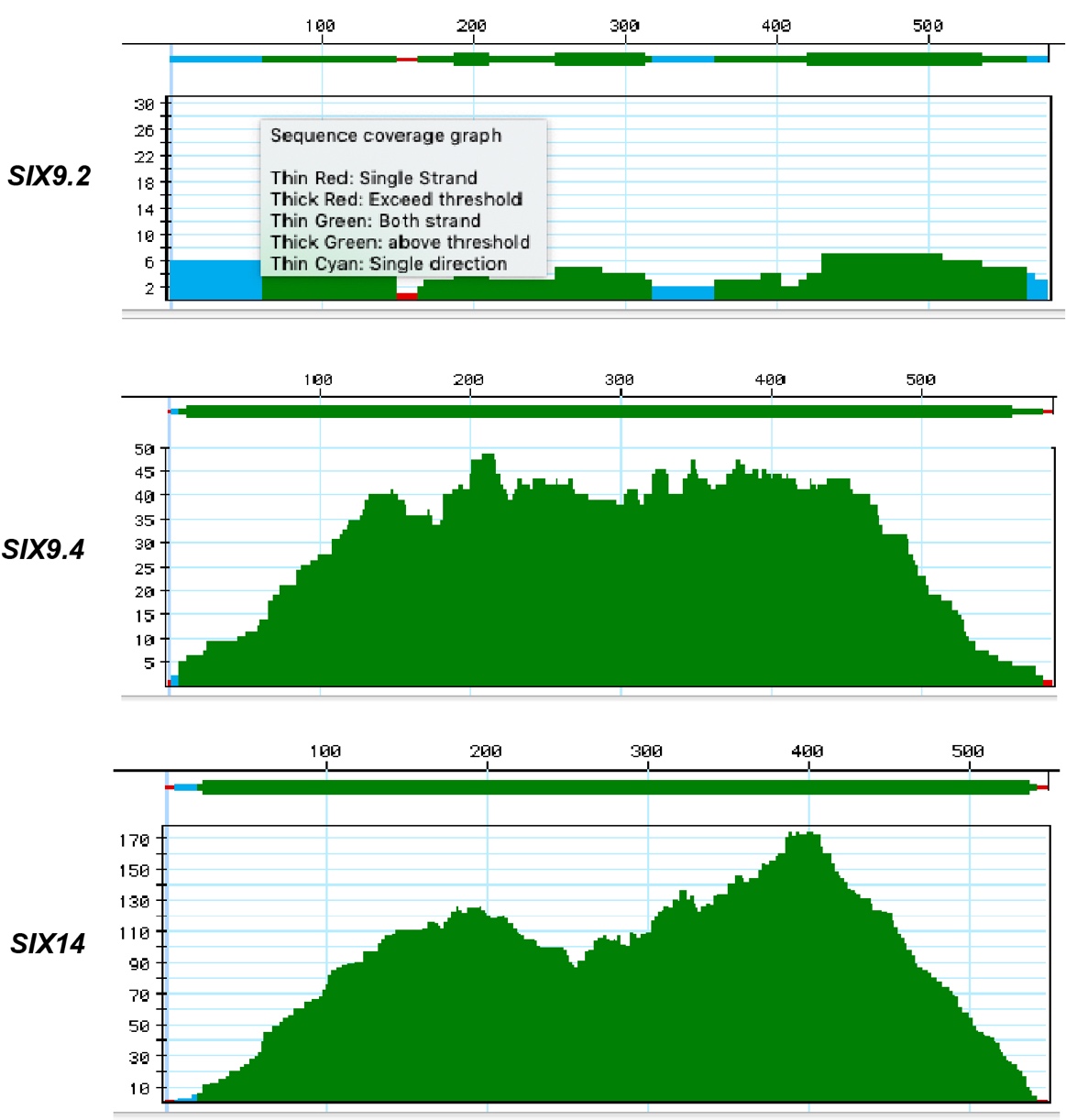


Supplementary Figure S8. Coverage of Illumina reads from AM163 (FOLac race 1) mapping to the reference sequences of *SIX9.2*, *SIX9.4*, and *SIX14*. Mapped reads that were one-directional and both-directional were indicated in blue and green lines, respectively. Red lines indicate regions where only one read was mapped. Detailed description of the coverage graph is shown in the box inserted in *SIX9.2* read mapping graph. Coverage threshold is set to 2×.


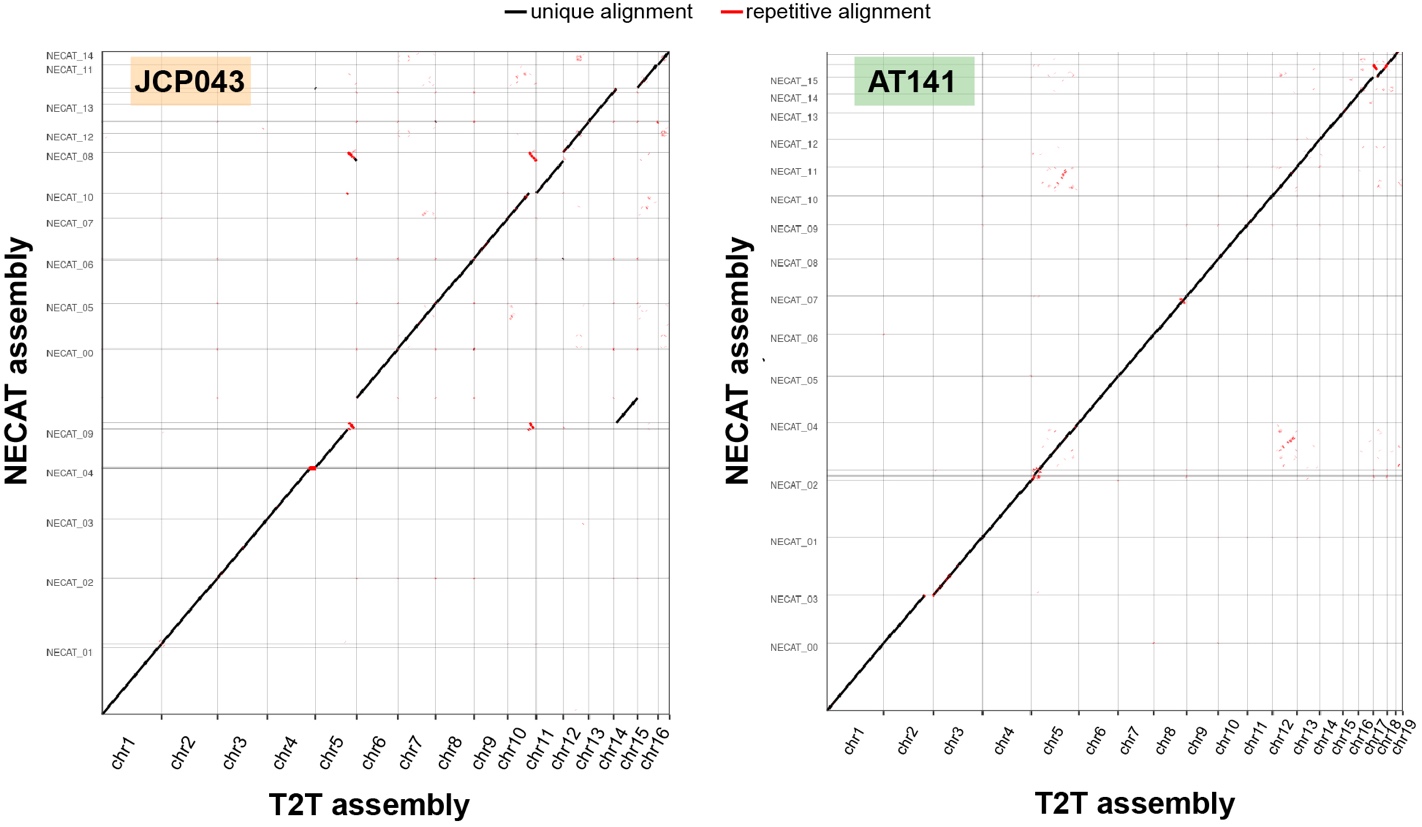


Supplementary Figure S9. Dot plots depicting the synteny between the T2T and NECAT assemblies of (A) JCP043 and (B) AT141. The nucmer package (nucmer –maxmatch; –L 10000) was used to identify highly similar regions, followed by data visualization with Assemblytics.
